# Early exposure to phosphorus starvation induces genetically determined responses in *Sorghum bicolor* roots

**DOI:** 10.1007/s00122-024-04728-4

**Published:** 2024-09-11

**Authors:** Erick O. Mikwa, Benjamin Wittkop, Steffen M. Windpassinger, Sven E. Weber, Dorit Ehrhardt, Rod J. Snowdon

**Affiliations:** Department of Plant Breeding, Justus Liebig University, Giessen, Germany

## Abstract

**Key message:**

We identified novel physiological and genetic responses to phosphorus starvation in sorghum diversity lines that augment current knowledge of breeding for climate-smart crops in Europe.

**Abstract:**

Phosphorus (P) deficiency and finite P reserves for fertilizer production pose a threat to future global crop production. Understanding root system architecture (RSA) plasticity is central to breeding for P-efficient crops. Sorghum is regarded as a P-efficient and climate-smart crop with strong adaptability to different climatic regions of the world. Here we investigated early genetic responses of sorghum RSA to P deficiency in order to identified genotypes with interesting root phenotypes and responses under low P. A diverse set of sorghum lines (*n* = 285) was genotyped using DarTSeq generating 12,472 quality genome wide single-nucleotide polymorphisms. Root phenotyping was conducted in a paper-based hydroponic rhizotron system under controlled greenhouse conditions with low and optimal P nutrition, using 16 RSA traits to describe genetic and phenotypic variability at two time points. Genotypic and phenotypic P-response variations were observed for multiple root traits at 21 and 42 days after germination with high broad sense heritability (0.38–0.76). The classification of traits revealed four distinct sorghum RSA types, with genotypes clustering separately under both low and optimal P conditions, suggesting genetic control of root responses to P availability. Association studies identified quantitative trait loci in chromosomes Sb02, Sb03, Sb04, Sb06 and Sb09 linked with genes potentially involved in P transport and stress responses. The genetic dissection of key factors underlying RSA responses to P deficiency could enable early identification of P-efficient sorghum genotypes. Genotypes with interesting RSA traits for low P environments will be incorporated into current sorghum breeding programs for later growth stages and field-based evaluations.

**Supplementary Information:**

The online version contains supplementary material available at 10.1007/s00122-024-04728-4.

## Introduction

Sorghum (*Sorghum bicolor* (L.) Moench) is the fifth most important and most climate resilient of the major carbohydrate-rich cereals crops (Hossain et al. 2022). Future cropping predictions under expected climate scenarios depict a likely increase in sorghum production alongside a 5–25% decline in other starch crops (Teferra and Awika 2019). The potential of sorghum as an alternative to maize (*Zea mays*) is widely accepted (Windpassinger et al. 2015; Ahmad Dar et al. 2018; Stamenković et al. 2020). Over 60 thousand metric tons of sorghum was produced in 2022, of which Africa, Asia and Latin America contributed about 70% (USDA 2022). Irrespective of the low production in Europe (1%), high demands have been observed with more than double production increase in the last decade (FAO 2022). However, sorghum production is not only below its potential but also endangered by unpredictable climate change and soil nutrition dynamics.

Soil nutrient depletion threatens sustainable global food security (Smith and Gregory 2013; Kopittke et al. 2019). Nutrient deficiencies caused by the continuous pressure on agricultural land and disruption of natural ecosystems by increasing human population can no longer be ignored (Kopittke et al. 2019; Sarkar et al. 2020). Major sorghum-producing countries, especially those in the tropics, experience significant soil P deficiencies. P deficiency and its global budget as a key fertilizer component also threatens food production in many countries (Zhang et al. 2020; Alewell et al. 2020). While it is required by crops in low concentrations, many reports indicate that over 40% of world soils are P deficient (Tiessen 2008; Obersteiner et al. 2013; Cordell and White 2015; Nedelciu et al. 2020; Alewell et al. 2020). Globally, P deposits are estimated to be depleted by around 40–100 years and will either be expensive or not attainable for low-income countries (Obersteiner et al. 2013; Cordell and White 2015). Paradoxically, high-income countries contend with the continuous eutrophication of freshwater bodies due to P pollution from the fields (Cordell et al. 2009; Obersteiner et al. 2013). Hence, emerging sorghum-producing countries in Europe, where fertilizer supply is sufficient, are considering breeding as an alternative to prevent P pollution (Nedelciu et al. 2020; Alewell et al. 2020; Han et al. 2022).

Soil P availability to plants is unlike other macronutrients due to its immobility and sorption properties to other elements (Larsen 1967; Richardson et al. 2009; Shen et al. 2011). Plants take up P in the form of orthophosphates (Pi) from various sources, both organic and inorganic (Lambers 2022). Organic P must be mineralized before uptake (Vance et al. 2003) while inorganic P (from commercial fertilizers) comes in different forms (Syers et al. 2008; Tiessen 2008). Plants prioritize P in the soil solution before exploiting P adsorbed on soil surfaces (Lambers 2022). The strong phytate bonds with calcium, iron or aluminum in many soil types also limit P to the top profiles rendering it unavailable for root systems spanning lower soil profiles (Richardson et al. 2009). As a result, P-efficient crops exhibit RSA characterized by elongated lateral roots distributed within the upper soil profiles. Moreover, most plants employ various mechanisms to enhance phosphate acquisition efficiency (PAE). These include increased density of lateral roots and root hairs at the expense of basal roots, widened angles between lateral roots and primary roots, enhanced solubilization at the soil surface, and improved interactions with beneficial microbes, among other strategies (Lynch and Brown 2001; Liu 2021; Han et al. 2022). Likewise, cellular and molecular stimuli of many crops are predisposed by such RSA adaptations under heterogeneous soil P profiles and regional deficiencies (Rogers and Benfey 2015; Ge et al. 2019; Maqbool et al. 2022). For instance, the vacuole pumps are activated to mobilize Pi from old to new leaves (Hammond et al. 2004; Veneklaas et al. 2012). Upon depletion without external supply, plants tend to scavenge from organic P such as lipid P, ester-P and nucleic P to maintain growth and development (Veneklaas et al. 2012; Han et al. 2022). Early detection of RSA plasticity under varying P conditions is key toward breeding for P-efficient crops.

Recently, the organization of different root types (RSA) has become a major crop breeding target due to its untapped significance in nutrient uptake efficiency and potential impact on yield (Zobel and Waisel 2010; Kuijken et al. 2015; Lynch 2019; Schneider and Lynch 2020). Many studies have identified genes corresponding to the overall impact on P stimulus and how they influence particular root and shoot traits, especially in rice (*Oryza sativa*) and arabidopsis (*Arabidopsis thaliana*) (Han et al. 2022). Breeders can also take advantage of the well-established diversity and heritability of the RSA to discover QTL and genes associated with root traits for efficient nutrients uptake (Schneider and Lynch 2020). Several QTL associated with P acquisition (PA) and/or utilization (PU) efficiency and yield related traits have been discovered in cereal crops such as finger millet (*Eleusine coracana (L.) Gaertn*.) (Maharajan et al. 2023), wheat (*Triticum aestivum*) (Yuan et al. 2020), soybean (*Glycine max*) (Gao et al. 2020) under low and high P conditions. As a result, genes encoding proteins such as Pi transporters, acid phosphatases, and DNA repair proteins have been identified. However, only a few related genes associated with RSA have been discovered in sorghum under contrasting P conditions.

Previous studies have revealed the potential of sorghum RSA plasticity for selection of improved varieties. For instance, Singh et al. (2011) observed moderately high heritability (0.47) in nodal root angle with an independent variation to plant size recommended for screening drought tolerant inbred lines and hybrids. Likewise, advanced genomics studies and availability of mapping populations has inspired gene discovery through QTL mapping, transcriptomics and genome wide association studies (GWAS) in sorghum (Zhang et al. 2019; Hao et al. 2021). Recently, using two association panels (Hufnagel et al. 2014) shown that rice protein kinase, *PHOSPHORUS-STARVATION TOLERANCE1* (*OsPSTOL1*) performed a more general role of increasing both yield and RSA in sorghum under low P hydroponic nutrient conditions. Similarly, QTL encoding rice homologs serine/threonine receptor kinase and *OsPSTOL1* were associated with enhanced root morphology and grain yield under low P conditions in sorghum (Parra-Londono et al. 2018; Bernardino et al. 2019).

Nevertheless, only a limited number of studies have attributed a significant level of importance to morphological and molecular adaptability of RSA under low P in sorghum. Additionally, the challenges posed by variability in root morphology and the complexities associated with root phenotyping have hindered exploration of the genetic basis underlying RSA plasticity in response to phosphorus (P) deficiency. We suggest that a precisely designed high-throughput hydroponic experiment can accelerate the early detection of heritable variations in sorghum under P-deficient conditions. This study aimed at determining phenotypic variation and diversity of sorghum RSA ideotypes and detecting early genetic responses of sorghum RSA under low P conditions.

## Materials and methods

### Germplasm

Diverse sorghum lines (*n* = 285) representing all sorghum races (*bicolor, caudatum, durra, guinea, and kaffir*) and their intermediate sub-races were used in this study. About 44% (*n* = 126) of the population consisted of *breeding lines* from a joint breeding program of Justus Liebig University Giessen, Norddeutsche Pflanzenzucht Hans-Georg Lembke KG (Hohenlieth, Germany) and Deutsche Saatveredelung AG (Lippstadt, Germany). Early maturing sorghum *conversion lines* comprised about 33% (*n* = 94) of the population. The remaining 23% (*n* = 65) consisted of publicly available *genebank accessions* from temperate countries (mainly China, Russia and the USA), but most of the seeds were obtained from the *United States Department of Agriculture Agricultural Research Service* (USDA-ARS) (Supp. Table 1).

### Greenhouse experimental set up

The experiments were conducted in the greenhouse at Justus Liebig University, Giessen in three successive, independent replications in summer 2022. For each experiment, a greenhouse chamber with free air circulation, approximately 65% humidity and a temperature of 28/24 °C (14 h day/10 h night) was used. To create a paper pouch, a 5-mm-thick and 1-cm-wide foam mattress (Polyetherpaleis, The Hague, Netherlands) was stapled along the edges of blue germination paper (25 × 30 cm) (Anchor paper Co., St. Paul, MN, USA). A piece of 595_1/2_ folded filter paper (Ø 125 mm) (Schleichner & Schnell, Dassel, Germany) cut into single wedges was then attached between the foam and the germination paper at the top center position, and covered with a black polythene paper (25 × 30 cm) (Supp. Figure 1a and b). After sterilizing using 3% NaOCl for 15 min two seeds (thinned to one three days after germination) were sown approximately 1.5–2 cm from the top covered by the folded filter paper wedge. Ten pouches were sandwiched using Plexiglas, tied with rubber bands (220 × 6 mm) (Alco-Albert, Arnsberg, Germany) then dipped in contrasting optimal and P-deficient nutrient solutions to a height of 14 cm in a 42 L Eurobox (60 × 40 × 22 cm) (Surplus Systems, Bonn, Germany) nutrient boxes (Supp. Figure 1c). A total of 120 genotypes were stacked in each box containing 10.5 L of nutrient solution in completely randomized design in three replicates. The following nutrient concentrations were used in optimal conditions: 472.3 mg/L Ca(NO_3_)_2_·4H_2_O, 278.8 mg/L K_2_SO_4_, 184.5 mg/L MgSO_4_·7H_2_O, 27.2 mg/L KH_2_PO_4_, 7.5 mg/L KCl, 73.4 mg/L FeEDTA, 0.034 mg/L MnSO_4_·H_2_O, 0.144 mg/L ZnSO_4_·7H_2_O, 0.045 mg/L CuSO_4_·5H_2_O, 0.012 mg/L (NH_4_)_6_Mo_7_O_24_·4H_2_O, and 0.006 mg/L H_3_BO_3_ to a P concentration of 6.19 mg/L. Alternatively, the P-deficient nutrient solution (0 mg/L) was prepared to compensate for P after KH_2_PO_4_ exclusion by adjusting the concentration of K_2_SO_4_ to 261.4 mg/L. The nutrient solution was maintained under continuous aeration using 12,000 L capacity ventilation pumps (Heissner, Lauterbach, Germany) with the pH maintained between 5.9 and 6.1 replaced after every five days.

### Image acquisition and trait extraction

Images were captured using Canon EOS 1000D camera (Cannon, Krefeld, Germany) mounted on a stand above a photo light box (Yorbay, Harmburg, Germany) (Supp. Figure 1d). The paper pouches with seedlings were laid one at a time in the light box and images taken through a hole from the top. All the images were captured at a resolution of 353 dpi 21 days after germination (DAG) and 42 DAG for both P-deficient and optimal conditions. The camera was connected to a computer and images captured using default camera settings. At 42 DAG, shoots and roots were harvested and oven-dried at 70 °C for 3 days before weighing. A root-shoot ratio was calculated, defined as the logarithm of the ratio between the root dry weight (RtW) and the total plant dry weight (Tw) according to the method described by Zobel and Waisel (2010). Upon careful consideration, it was proposed that deriving the ratio from the logarithm of root weight to the logarithm of the total plant weight (Tw) would provide a more meaningful interpretation compared to the conventional root-to-shoot ratio. The Tw was calculated from the sum of RtW and shoot dry weight (ShtW). For the other RSA traits, 2D images (Supp. Figure 1e and f) were first processed using imageJ software to a low pixel gray background format (Schneider et al. 2012; Borianne et al. 2018). All images were first batch processed by background subtraction with the following options: light background, disabled smoothing and a rolling ball of 500 pixels. The segmentation process was performed using the triangle thresholding method with red, green and blue (RGB) color space set at minimum–maximum pixels of 115–255, 0–255, and 255–255, respectively.

**Fig. 1:**
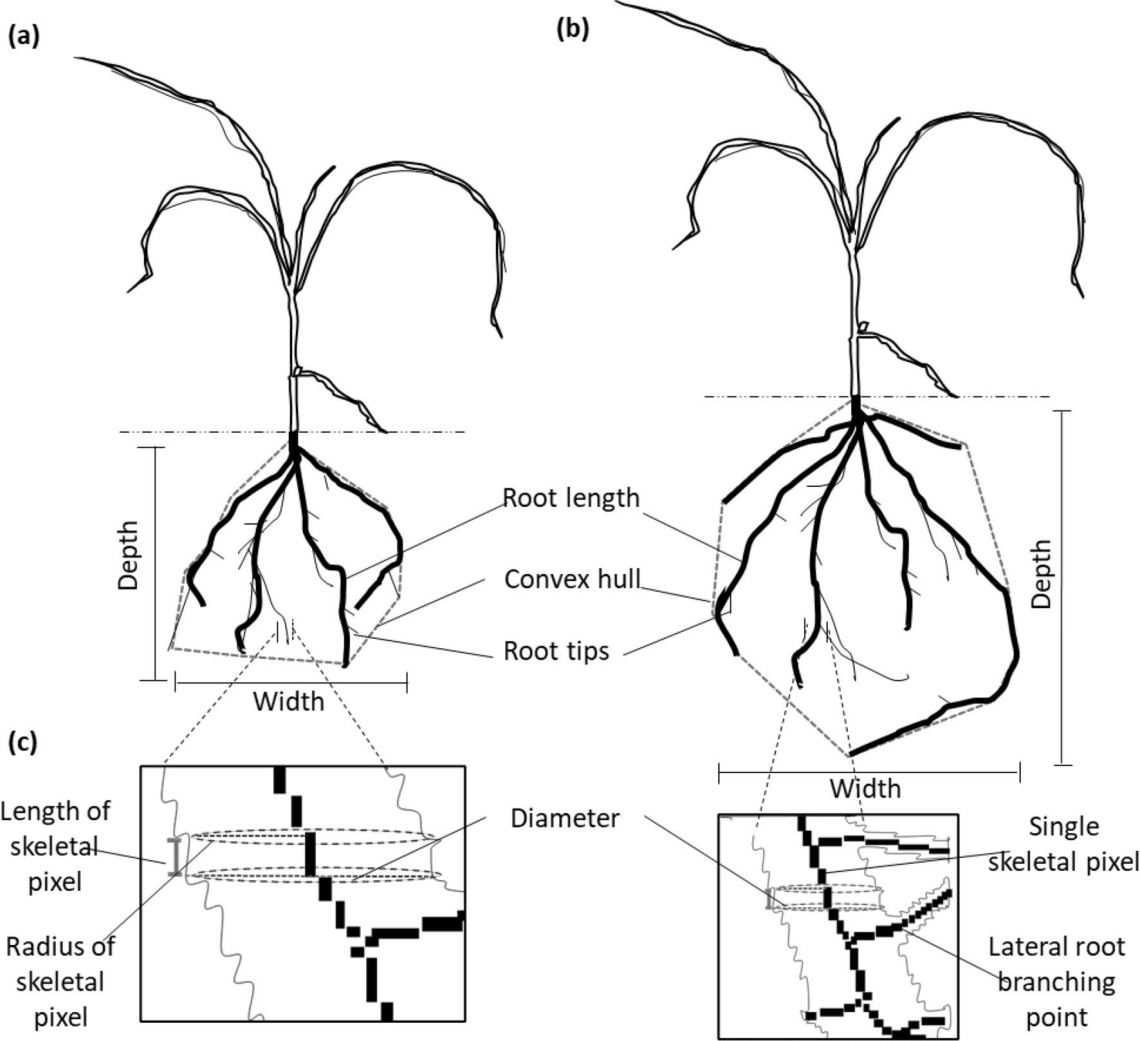
A diagram of RSA traits as extracted by RhizoVision explorer (Seethepalli et al. 2021) **a** A representation of RSA under optimal P conditions showing shorter root length, convex area and fewer root tips but larger root diameter **b** A representation of RSA under P-deficient conditions showing longer root network, convex area, more root tips, but thinner diameter **c** Extended root cross-section as depicted by RhizoVision explorer. An overall smaller network area, volume and surface area characterize RSA under optimal P conditions as compared to P-deficient condition with larger root network but thinner diameters

RhizoVision explorer software v2.0.3 was used to extract the RSA traits of interest (Seethepalli et al. 2021). Images were first pre-processed using whole root settings and pixels converted to physical units (mm) at 353 dpi per mm. Non-root objects were filtered at 5 pixels, non-root objects set at maximum size of 3 mm^2^ and edge smoothing set to 2 pixels. Feature extraction for agronomic traits involved converting raw pixels into numeric representations, utilizing a root pruning threshold of 3 pixels and setting root diameter ranges of 0–2 mm, 2–5 mm, and 5–6 mm. The diameter ranges were chosen carefully in consideration of the first and second order of lateral roots (Seethepalli et al. 2021). The following traits were assessed from RhizoVision: Maximum number of roots (MaxnR), number of root tips (noRtips), total root length (TrLngth) (mm), root depth (mm), maximum root width (MaxWdth) (mm), network area (NetA) (mm^2^), convex area (ConA) (mm^2^), solidity (Soldty), average root diameter (AvDim) (mm), perimeter (Per), volume (Vol) (mm^3^), surface area (SurfA) (mm^2^), and average root orientation (AvOrnt) (degrees) (Fig. 1). Detailed description of the chosen traits has been defined by Seethepalli et al. (2021).

### Phenotypic analyses

For each time point (21 DAG and 42 DAG), analysis of variance (ANOVA) was fitted using linear mixed model in R, where factors individual genotypes and nutrient conditions, optimal P and P deficient were considered as fixed effects while, genotype-nutrient interactions, replicates, nutrient containers/boxes, column and row position of sandwiched paper pouches in the boxes were considered as random effects (Bates et al. 2015; R Core Team 2023). Statistical difference for the ANOVA was assumed at *P* < 0.05 using the following model:

1 $$Y_{ijklmo} = \, \mu \, + \, G_{i} + \, E_{j} + {\text{ GE}}_{ij} + r_{kj} + b_{lmo} + c_{m} + r_{o} + \, \varepsilon_{ijklmo}$$

where *Y*_*ijklmo*_ is the phenotypic values of genotype *i* (*i* = 1…*n*_*g*_) in the nutrient condition *j,* replicate *k,* nutrient box *l,* column *m* and row *o*; *μ* represents the population mean; *E*_*j*_ is the environmental effect, GE_*ij*_ is the genotype-by-nutrient condition interaction, *r*_*kj*_ is the replicate effect, *b*_*lmo*_ nutrient box effect, *c*_*m*_ column effect *r*_*o*_ row effect, and *ε* is the residual effect. The data were analyzed in R using lmer package (Bates et al. 2015).

Broad sense heritability (*H*^*2*^) was estimated for each trait and condition according to Piepho and Möhring (2007).

2 $${H}^{2}=\frac{{\sigma }_{G}^{2}}{{\sigma }_{G}^{2}+\frac{\underset{\_}{\overline{\text{v}}}}{2} }$$

where ${\sigma }_{G}^{2}$ is the genotypic variance obtained from a full random model as described above, $\overline{\text{v} }$ is the mean variance of all genotype contrasts.

To determine significant difference between the two growing conditions and time points, Student–Newman–Keuls post hoc-test was applied to the within environments ANOVA results (Abdi and Williams 2010). For each condition per time point, adjusted means were estimated taking genotype effect as fixed factor while, replicate, nutrient box, column and row as random effects using the following generalized linear model:

3 $$Y_{ij} = \, \mu \, + \, G_{i} + r_{j} + \, b_{lmo} + \, c_{m} + \, r_{o} + \, \varepsilon_{ijlmo}$$

where *Y*_*ij*_ is the phenotypic values of genotype *i* (*i* = 1…*n*_*g*_) in the nutrient condition *j*; *μ* represents the population mean; *G*_*i*_ is the genotypic effect per condition, *r*_*j*_ is the replicate effect per condition, *b*_*lmo*_ nutrient box effect, *c*_*m*_ column effect *r*_*o*_ row effect, and *ε* is the residual effect. The adjusted means (Supp. Table 5 and 6) were used to compute within time point means and standard deviation, principal components analysis, correlations, and relative differences between the contrasting nutrient conditions. The relative difference (Supp. Table 7) was estimated for each trait using the optimal conditions as the comparison indicator according to (Lopez et al. 2023):

4 $${\text{Relative difference }} = { }\frac{{\overline{x}_{0} - \overline{x}_{1} }}{{\overline{x}_{1} }}$$

where $\overline{{x }_{0}}$ is the adjusted means for the traits with P deficient while $\overline{{x }_{1}}$ represents adjusted means of the traits with optimal nutrient condition. Pearson’s correlation (*r*) was estimated in R comparing all traits at each time point and condition.

### Root system architecture classification analysis

Sorghum root system classification was performed according to Bodner et al. (2013). The classification relies on multiple topological traits and is executed without prior cluster information about the genotypes. Each principal component captures a particular group of traits and in between tradeoffs that when put together defines an RSA type. All traits were subjected to principal components analysis (PCA) and cluster analysis using R (R Core Team 2023). Principal components were computed from scaled means in R package FactoMiner (Lê et al. 2008) and bi-plots plotted using factoextra package (Kassambara and Mundt 2020). Cluster analysis was done using agglomerative hierarchical with hclust and ward method in R (Murtagh and Legendre 2014) for each time point and condition. The classification of the RSA was based on the vertical distribution of roots and branching intensity, following the approach outlined by Bodner et al. (2013). Clusters were used to identify four architectural groups, described by Degens (1997) based on differences in the distribution of organic carbon inputs. The architectural groups include fine root, coarse root, restricted root branching and highly branching root systems. The groups were ranked by density and distribution from small to large root systems, successively. PCA bi-plots for optimal conditions were used as a base for classifying for P-deficient conditions. Boxplot functions in R were used to determine phenotypic differences based on the major clusters.

### SNP genotyping and quality control

Genomic DNA extracted from seedling leaves of the diversity set were genotyped by the service provider Diversity Arrays Technology Pty Ltd., Canberra, Australia (www.diversityarrays.com) using the proprietary DArTSeq Sorghum genotyping service. DarTSeq is a hybridization-based genotyping platform that uses restriction enzymes to target the most variable (predominantly active) regions of the genome before sequencing. SNP calling used the *Sorghum bicolor* reference genome version v3.1.1 (McCormick et al. 2018). A total of 31,732 raw markers were called, which generated over 12,472 genome wide SNPs after quality filtering. Quality SNPs were retained based on missing marker information (less than 70%), minimum allele frequency (less than 5%), and heterozygosity (at least 10%).

### Population structure and diversity analysis

Population structure was inferred using both STRUCTURE software 2.3.4 and discriminant analysis of principal components (DAPC) (Pritchard et al. 2000; Jombart et al. 2010). The STRUCTURE run was done in a bunning period of 50,000 iterations and 100,000 Markov Chain Monte Carlo (MCMC) repeats after burning with 1 to 10 iterations assuming the admixture model. Races/genotype sources were used as prior information for the population. In addition, population structure was determined by DAPC using the R package adegenet (Jombart et al. 2011; R Core Team 2023). Unlike PCA which infers global diversity and tends to overlook the between groups variability, DAPC analysis uses PCA to minimize within group variability and maximized across group variability (Jombart et al. 2010).

### Linkage-disequilibrium decay and SNP density distribution

Linkage-disequilibrium (LD) decay was measured using *r*^2^ with distance in Kilobases in R. Before plotting, loess smoothing was fitted on the mean LD values per chromosome with a smoothness factor of 0.005 for the curve and 4000 evaluation points (Jacoby 2000). Population variability during window selection was corrected using a kinship matrix generated from GeneAbel (Aulchenko et al. 2007). Whole-genome SNP density distribution was computed based on the filtered SNPs using CMplot package with a window size of 1 Mb (Yin et al. 2021).

### Genome wide association studies and haplotype analysis

Genome wide association study was conducted on the 12 k SNP panel with 285 genotypes and means of RSA traits using genome association and prediction integrated tool (GAPIT) (version 3) incorporated in two models (Wang and Zhang 2021). The models used include fixed and random model circulating probability unification (FarmCPU) (Liu et al. 2016) and Bayesian information and Linkage-disequilibrium Iteratively Nested Keyway (BLINK) (Huang et al. 2019). The first three principal components were fitted as covariates to reduce false positives due to population stratification (Wang and Zhang 2021). Both multi-loci models (FarmCPU and BLINK) analyses were performed to validate significant QTL and identify rare peaks (Neupane et al. 2023). Only significant QTL (at corrected FDR < 0.05) that were found within the sorghum average LD decay cut-off with LD blocks as established by Haploview version 4 were considered (Gabriel et al. 2002; Barrett et al. 2005). Candidate genes were identified based on sorghum reference v3.1.1 from phytozome v13 within the confidence intervals of haplotype blocks harboring significant SNPs (McCormick et al. 2018; Ugwuanyi et al. 2022).

Haplotype screening was performed by an initial identification of SNPs within the LD blocks using Haploview version 4, as defined by Gabriel et al. (2002). Haplotypes were then identified within a block distance, selected based on the global sorghum 50% LD decay within 150 kb (Morris et al. 2013). The selected haplotypes with a minimum allele frequency of 0.05 were then utilized to categorize phenotypic data within our population (Chakrabarthy et al. 2021; Achola et al. 2023). To determine significant differences (*p*-value < 0.05) among the means of haplotypes associated with corresponding traits, one-way ANOVA was conducted. In addition, boxplots were employed to visualize of haplotype effects on associated traits (R Core Team 2023).

## Results

### Multivariate analysis reveals varying responses RSA to P availability

We conducted a multivariate analysis of P-related traits (i.e., traits that are influence by P availability) in sorghum’s RSA under varying P conditions and time points. P-response variations were evident in several root-related traits as early as 21 DAG (Table 1). The coefficient of variation (CV) ranged from 8.7 to 59.7% under optimal phosphorus and from 8 to 69% under P-deficient conditions. A higher degree of variation was observed under P-deficient conditions in most traits as compared to optimal conditions, indicating a stronger influence of P deficiency on the population. Root system architecture traits, such as SurfA, TrLngth and NetA, showed the highest CV at each condition and also demonstrated significant differences in means between the two conditions (*α* < 0.05) (Table 1). Besides, there was a significant interaction between genotypes and nutrient supply for these traits accompanied by high heritability.

**Table 1:** Analysis of variance and broad sense heritability for each condition and time point

Traits	Optimal phosphorus			Phosphorus deficient			G	P	G*P	H^2^
	Mean	S.d	%Cv	Mean	S.d	%Cv				
*21 Days after germination*										
MaxnR	24.24	8.01	33	26.01	8.75	33.6	***	***	*	0.59
noRtips	181.54	83.71	46.1	207.07	103.39	49.9	***	***	***	0.73
TrLngth (mm)	241.63	118.54	49.1	291.02	156.71	53.8	***	***	**	0.73
Depth (mm)	20.64	2.89	14	22.77	3.25	14.3	***	***	ns	0.50
MaxWdth	13.42	3.52	26.2	13.70	3.51	25.6	***	ns	ns	0.62
NetA (mm^2^)	10.50	5.03	47.9	14.04	7.16	51	***	***	***	0.71
ConA (mm^2^)	184.53	69.29	37.5	206.81	76.96	37.2	***	***	ns	0.69
Soldty	0.06	0.02	36.4	0.07	0.02	25	***	***	ns	0.38
AvDim (mm)	0.06	0.01	13.3	0.06	0.01	11	***	***	ns	0.58
Per (mm)	429.57	203.21	47.3	501.50	257.66	51.4	***	***	**	0.73
Vol (mm^3^)	0.77	0.46	59.7	1.23	0.85	69	***	***	***	0.59
SurfA (mm^2^)	41.39	20.86	50.4	56.88	31.30	55	***	***	***	0.69
AvROrnt (deg)	47.01	4.07	8.7	46.29	3.68	8	***	**	ns	0.47
*42 days after germination*										
MaxnR	36.01	9.82	27.3	38.03	9.76	25.7	***	***	*	0.44
noRtips	265.72	102.00	38.4	306.17	113.73	37.1	***	***	**	0.64
TrLngth (mm)	392.76	188.72	48	451.85	184.15	40.8	***	***	**	0.65
Depth (mm)	19.56	2.75	14.1	21.40	2.65	12.4	***	***	ns	0.42
MaxWdth	14.27	2.82	19.8	14.50	2.74	18.9	***	ns	ns	0.48
NetA (mm^2^)	18.34	9.61	52.4	20.96	9.05	43.2	***	***	*	0.65
ConA (mm^2^)	184.88	56.36	30.5	206.06	57.13	27.7	***	***	ns	0.54
Soldty	0.10	0.03	34.2	0.10	0.03	25.1	***	ns	ns	0.44
AvDim (mm)	0.06	0.01	14.9	0.06	0.01	10.8	***	ns	*	0.30
Per (mm)	640.07	273.14	42.7	742.52	282.30	38	***	***	**	0.64
Vol (mm^3^)	2.33	2.21	94.6	2.33	1.42	60.8	***	ns		0.54
SurfA (mm^2^)	82.55	50.21	60.8	92.34	43.40	47	***	***	*	0.63
AvROrnt (deg)	46.49	2.74	5.9	46.27	2.73	5.9	***	ns	ns	0.40
ShtW	0.06	0.03	44.4	0.07	0.03	43.5	***	***		0.76
RtW	0.04	0.02	42.2	0.04	0.02	45.5	***	***	**	0.54
RtW:Tw	1.42	0.11	7.8	1.47	0.13	8.7	***	***	ns	0.76

Significance codes: 0 ‘***’ 0.001 ‘**’ 0.01 ‘*’ 0.05 ‘•’ 0.1 ‘ns’; Standard deviation (S.d), coefficient of variation percentage (% Cv), genotype effect (G), phosphorus treatment effect (P), genotype by P treatment effect (G*P), broad sense heritability (H^2^), dry shoot weight (ShtW), dry root weight (RtW), maximum number of roots (MaxnR), number of root tips (noRtips), total root length (TrLngth), maximum width (MaxWdth), network area (NetA), convex area (ConA), solidity (Soldty), average diameter (AvDim), perimeter (Per), volume (Vol), surface area (SurfA), average root orientation (AvOrnt), log_10_(dry root weight/total plant weight) (Rtw:Tw)

Successively, CV ranged from 5.9 to 94.6% under optimal P and from 5.9 to 60.8% under P-deficient conditions at 42 DAG. At this time point, higher CV was observed under optimal conditions for most traits in contrast to P-deficient conditions. Nevertheless, significant differences (*α* < 0.05) in means were observed for genotypes and genotype-nutrient interactions in P-related RSA traits such as noRtips, TrLngth, NetA, and SurfA. The broad sense heritability for the traits ranged from 0.3 to 0.76, with the highest values observed in RtW:Tw at 42 DAG. Some of the traits with high heritability (> 0.6) at both time points included noRtips, TrLngth, NetA, Per, SurfA and ShtW. In contrast, traits such as AvOrnt and Soldty exhibited lower CV and heritability, indicating a weaker effect of the P treatment to these traits in the population. Despite the lower heritability observed at 42 DAG as compared to 21 DAG, significant differences (*α* < 0.05) in means were evident under both optimal P and P-deficient conditions for most of the traits (Table 1).

A comparison between the two time points revealed an observable increase in the overall mean for more than half of the measured traits, from 21 to 42 days (Supp. Figure 2). Significant differences in the mean were observed at both time points for traits such as noRtips, TrLngth, NetA, Per, and SurfA. At 21 DAG, all traits showed significant difference (*α* < 0.05) except MaxWdth (Supp. Figure 2). Although there were no significant differences in traits such as Vol, Soldty and AvDim at 42 DAG, significant differences between the two conditions were observed at 21 DAG (Supp. Figure 2). Traits describing root size, density, distribution displayed significant increases as compared to traits describing orientation and thickness.

A consistent pattern was observed for per trait correlations (*r*) between optimal P and P deficiency across the two time points (Supp. Figure 3). Significant correlations were evident at 21 DAG as compared to 42 DAG, with r values ranging from 0.31 to 0.69. Among the traits examined, TrLngth, ConA, Per, NetA and SurfA showed the highest correlation (*r* ≥ 0.6) at 21 DAG. In contrast, AvDim, Soldty, depth and AvOrnt exhibited the lowest correlation (*r* ≤ 0.45) at the same time point. Similarly, this trend was observed at 42 DAG, although with slightly lower r ranging from 0.25 to 0.58. Traits with the highest correlations averaged above *r* ≥ 0.56 while the lowest ranking traits showed correlations averaging below *r* ≤ 0.33 at 42 DAG. At this time point, the highest correlation (*r* = 0.7) was observed in dry shoot weight.

**Fig. 2:**
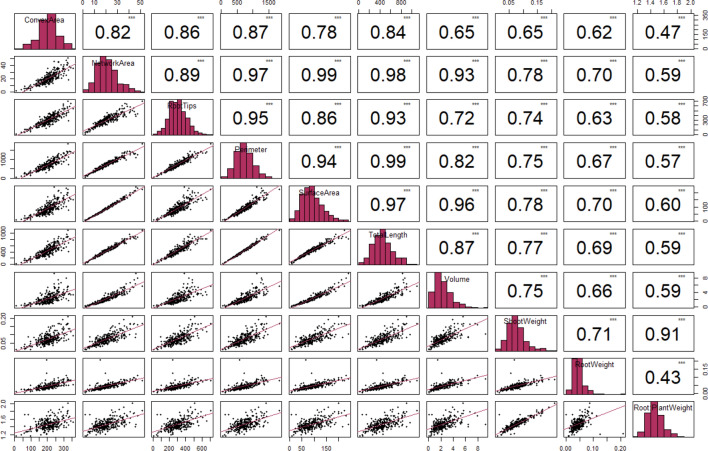
Pearson correlation matrix showing the correlation coefficient (*r*) and *p*-values of sorghum RSA traits that are positively associated with P-deficient conditions at 42 days after germination. Significance Codes: less than 0.01 ‘***’; greater than 0.01 ‘**’; greater than 0.1 ‘ns’

Positive and negative correlations were also observed among traits characterizing root size and distribution, in contrast to traits related to orientation and thickness, across various per time points and P treatment conditions. For instance, traits such as NetA and SurfA showed nearly perfect correlations (*r* = 0.99) in all time points and conditions, while strong positive correlations (*r* ≥ 0.9) were observed between pairs of traits such as TrLngth, NetA, noRtips, SurfA, Vol, MaxWdth, ConA, and Per across different conditions and time points (Supp. Figure 4–7). For these traits, the r values ranged from 0.90 to 0.97 under optimal P conditions and from 0.92 to 0.98 under P-deficient conditions at 21 DAG. At 42 DAG, similar strong positive correlations were observed for the above traits, with r values ranging from 0.86 to 0.98 under optimal P conditions and from 0.91 to 0.99 under P-deficient conditions. In contrast, traits such as Soldty, AvDim and AvOrnt exhibited negative correlations with most of the traits, with r values ranging from -0.0039 between Soldty and MaxWdth to − 0.59 between AvOrnt and MaxWdth (Supp. Figure 4–7). When comparing the top and bottom ranking traits at 42 DAG with total plant dry weight, significant (*p* < 0.01) negative and positive correlations were observed (Fig. 2). Highly P-responsive traits such as TrLngth, ConA, Per, NetA and SurfA displayed positive correlations with RtW and ShtW, while a negative correlation was observed in root orientation traits against the rest of traits.

**Fig. 3:**
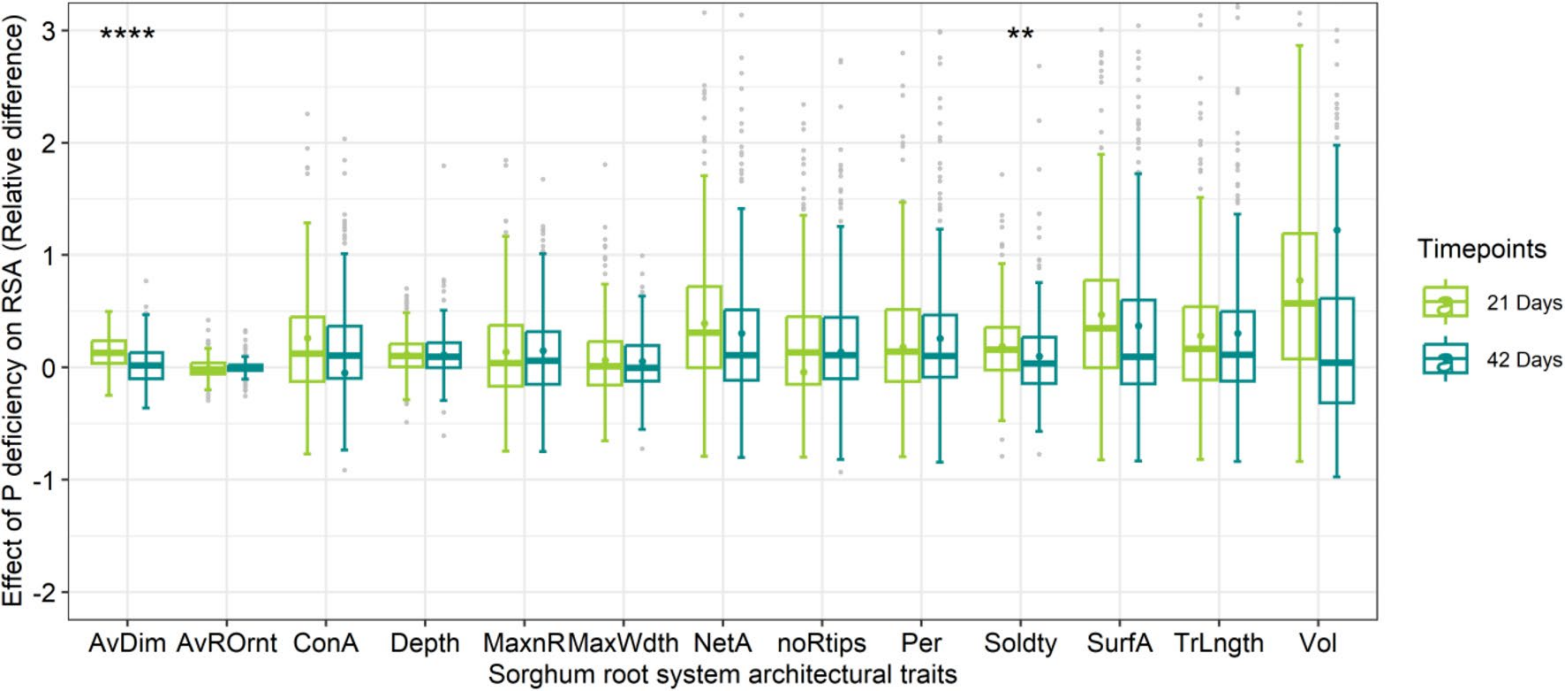
A plot of relative differences in sorghum root system architectural traits under optimal P and P-deficient conditions at 21 and 42 days after germination. The relative difference (RD) was obtained by the differences between adjusted means, from equation [4] above, of the two conditions and based on the optimal P condition RD = X0 − X1/X1. The mean comparisons between the two timepoints were performed using *t* test at 0.95 confidence level. Trait labels: dry shoot weight (ShtW); dry root weight (RtW); maximum number of roots (MaxnR); number of root tips (noRtips); total root length (TrLngth); maximum width (MaxWdth); network area (NetA); convex area (ConA); solidity (Soldty), average diameter (AvDim); perimeter (Per); volume (Vol); surface area (SurfA); average root orientation (AvOrnt)

Additionally, the relative difference between the two conditions revealed distinct variations in magnitude. The magnitude of change (median) was above 0 for all traits, except AvOrnt. Furthermore, a higher magnitude of difference was observed at 21 DAG as compared to 42 DAG in most of the traits, with the significant average differences observed in RSA such as AvDim and Soldty (Fig. 3).

### Phenotypic diversity clusters RSA across time points and conditions

**Fig. 4:**
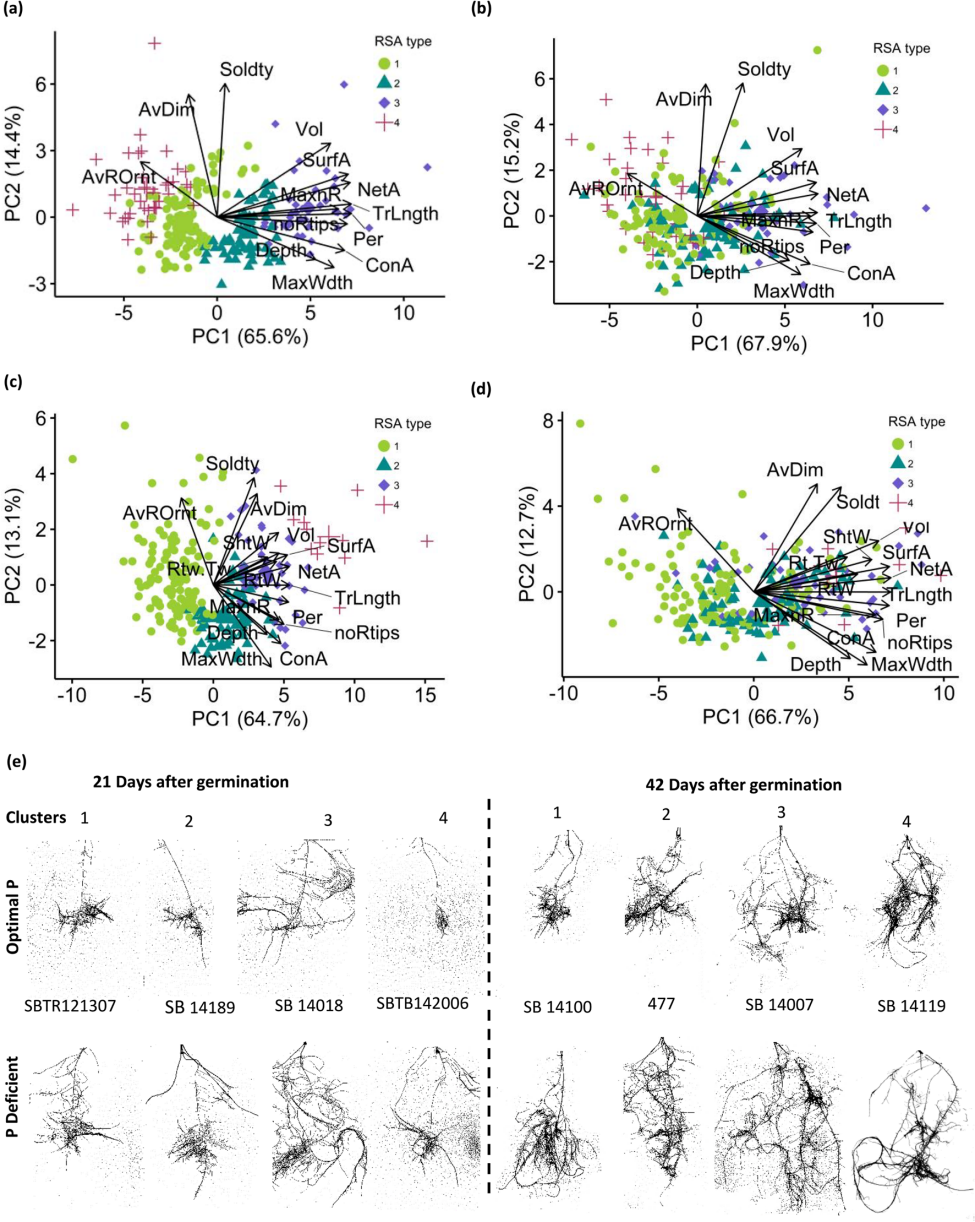
Phenotype-based sorghum RSA classification using PCA and root types using cluster analysis. The PCA bi-plot clusters of P-deficient condition was based on the optimal P clusters at each time point. **a–d** Bi-plots of optimal P at 21 DAG (**a**), P deficient at 21 DAG (**b**), optimal P at 42 DAG (**c**), and P deficient at 42 DAG (**d**), respectively **e** Root system architecture showing distinct root ideotypes at 21 and 42 days after germination (DAG) classifying diverse genotypes per P nutrient condition. Roots clusters at 42 DAG represents four architectural groups, namely highly branched (3), restricted (2), coarse (4), and restricted branched (1). Trait codes: average diameter (AvDim); average root orientation (AvOrnt); convex area (ConA); maximum number of roots (MaxnR); maximum width (MaxWdth); network area (NetA); number of root tips (noRtips); perimeter (Per); solidity (Soldty); surface area (SurfA); total root length (TrLngth); volume (Vol); dry shoot weight (ShtW); dry root weight (RtW); Log_10_(dry root weight/total plant weight) (Rtw:Tw)

The phenotypic diversity analysis using PCA bi-plots revealed distinct differences in sorghum RSA at each time point and under different nutrient conditions (Fig. 4). While different clustering was observed at 21 DAG and 42 DAG, all the genotypes were clustered into four ideotypes. At 21 DAG, the genotypes with the most significant changes under the two contrasting nutrient regimes were clustered in ideotype 3, whereas similar genotypes appeared in ideotype 4 at 42 DAG. Principal component 1 (PC1) displayed the highest loading at both time points and conditions, driven by several influential traits. Consequently, representative RSA ideotypes per cluster were chosen based on their contributions to the most influential variables in PC1 (Fig. 4e).

There was a positive influence of most traits to PC1, such as TrLngth, NetA, SurfA, and the noRtips at both time points (Fig. 4a–d). These traits positively contributed to PC1, while the main traits contributing to PC2 were AvDim, Soldty, and AvROrnt (Fig. 4a–d). Clusters 1 and 2 had relatively average contributions to the overall distribution of the population, suggesting a lower impact on the experiment. In contrast, clusters 3 and 4 classified genotypes with more distinct root ideotypes. These ideotypes were characterized as highly branched, restricted branched, coarse, and restricted most clearly visual at 42 DAG (Fig. 4e). Collectively, there was more common clustering between optimal P and P-deficient conditions at 21 days than at 42 days, with a similarity index of 0.78 and 0.48, respectively (Supp. Figure 8). The clusters described varying root distributions, density, and orientation.

### Genetic diversity and GWAS identifies SNPs across time points and conditions

The DarTseq technology generated a dataset of 31,732 SNPs, with 12,472 retained after quality filtering. In STRUCTURE analysis, optimal cluster patterns were observed at *K* = 3, *K* = 4, and *K* = 6 (Supp.Fig. 9a), providing insights into population structure and race distribution. Additionally, discriminant analysis of principal components (DAPC) identified four distinct components within the population (Supp.Fig. 9b), revealing the underlying genetic structure of individuals within our study. Furthermore, LD analysis showed an average LD decay of 45 kb across all chromosomes (Supp. Figure 9c), indicating a notable LD level over an extended physical range among the SNPs in the population.

Association studies revealed multiple SNPs at both time points and conditions, as well as relative differences (Table 2). In total, 4 QTL were discovered at 21 DAG, and 8 were identified at 42 DAG. These QTL were situated on chromosomes Sb02, Sb03, Sb04, Sb07, Sb08, Sb09, and Sb010, under both optimal P and P-deficient conditions. Higher numbers of QTL were associated with traits under P-deficient conditions compared to optimal conditions. Additionally, we identified a total of 15 SNP markers associated with relative differences at 21 DAG and 14 SNP markers associated with relative differences at 42 DAG, spread across chromosomes Sb01, Sb02, Sb03, Sb04, Sb06, Sb07, and Sb09 (Table 2). LD block sizes spanning these SNPs ranged from 0.044 to 75.4 kb when examining associations per time point and condition, and from 0.2 to 122 kb for relative differences (Supp. Table 2 and 3). Several pleiotropic SNPs were identified in multiple traits at both time points and conditions and relative differences (Table 2).

**Table 2:** Significant QTL under P-deficient conditions and relative differences between optimal P and P deficiency at 21 and 42 days after germination (DAG)

SNP	Alleles	Chr	Pos	MAF	Condition	Trait	Model	H&B.P.Value	PVE (%)	Effect
*Significant QTL associated with traits within the time points (21 day after germination)*										
S04_56068298	C/G	Sb04	56068298	0.25	OP	AvDim	FarmCPU	0.018856	3.67	− 0.002
S04_58029930	G/A	Sb04	58029930	0.07732	OP	AvDim	FarmCPU	0.018856	40.29	− 0.005
						AvDim	BLINK	7.74E-08	32.31	− 0.006
S04_5438539	G/C	Sb04	5438539	0.087629	DP	Depth	FarmCPU	0.000409	9.23	1.509
S09_59301045	C/T	Sb09	59301045	0.198454	DP	Per	BLINK	0.007894	6.99	118.918
						TrLngth	BLINK	0.004274	7.35	74.489
*Significant QTL associated with traits within the time points (42 day after germination)*										
S02_63147078	G/C	Sb02	63147078	0.113402	DP	AvROrnt	BLINK	0.003333	23.35	− 1.24
						ConA	BLINK	7.91E-08	17.31	34.5
						MaxWdth	BLINK	8.52E-07	14.09	1.56
						NetA	BLINK	0.00035	40.25	4.604
						noRtips	BLINK	1.98E-05	44.86	60.969
						Per	BLINK	4.87E-05	44.94	155.612
						TrLngth	BLINK	6.22E-05	44	101.156
						TrLngth	FarmCPU	0.002195	17.82	79.207
S03_66775420	A/C	Sb03	66775420	0.139175	DP	TrLngth	FarmCPU	0.005623	8.85	− 93.325
						Per	FarmCPU	5.29E-05	12.5	− 168.566
S03_781067	G/T	Sb03	781067	0.103093	DP	RtW:Tw	FarmCPU	2.83E-06	–	0.101
S04_60163766	A/T	Sb04	60163766	0.42268	OP	AvROrnt	BLINK	0.006717	8.58	− 1.099
S04_61445399	A/C	Sb04	61445399	0.074742	DP	ConA	BLINK	0.007903	19.9	− 39.3
S07_61184406	C/A	Sb07	61184406	0.180412	DP	ConA	BLINK	0.002126	8.36	28.294
S08_5681465	T/A	Sb08	5681465	0.247423	DP	MaxWdth	FarmCPU	0.010033	13.48	0.93
S10_55574810	G/T	Sb010	55574810	0.190722	DP	Vol	BLINK	0.01806	5.79	0.475
*Significant QTL associated with relative differences between the conditions based on the optimal P at 21 DAG*										
S02_4271214	A/G	Sb02	4271214	0.041237	RD	NetA	FarmCPU	3.9E-10	18.36	− 1.68
						Per	BLINK	1.45E-09	7.05	− 1.555
S02_4706323	T/C	Sb02	4706323	0.046392	RD	Per	BLINK	0.000703	3.44	− 0.783
S02_10586206	A/G	Sb02	10586206	0.5	RD	Per	BLINK	6.94E-07	0.14	− 0.524
S03_73478713	G/C	Sb03	73478713	0.095361	RD	ConA	FarmCPU	0.000154	7.56	− 0.826
						noRtips	FarmCPU	5.29E-07	5.89	3.503
						noRtips	BLINK	1.31E-06	1.14	2.6
S03_4443037	C/A	Sb03	4443037	0.087629	RD	TrLngth	FarmCPU	9.04E-06	12.83	− 0.837
S04_6401545	G/A	Sb04	6401545	0.03866	RD	noRtips	BLINK	0.001529	1.61	1.887
S06_51947681	A/G	Sb06	51947681	0.056701	RD	ConA	BLINK	3.55E-08	2.86	0.687
						ConA	FarmCPU	0.000451	7.12	0.584
S06_39982967	G/A	Sb06	39982967	0.028351	RD	NetA	FarmCPU	0.005061	9.27	− 1.008
S06_48113932	G/T	Sb06	48113932	0.095361	RD	Per	BLINK	0.00018	0.56	− 0.842
S07_1891754	A/G	Sb07	1891754	0.438144	RD	ConA	BLINK	0.001846	0.13	0.216
S09_58006574	G/A	Sb09	58006574	0.028351	RD	ConA	BLINK	2.86E-15	13.83	− 1.633
						ConA	FarmCPU	4.96E-05	11.65	− 1.222
						NetA	BLINK	9.2E-14	14.67	1.835
						noRtips	BLINK	1.02E-32	25.69	9.45
						noRtips	FarmCPU	1.9E-11	24.41	6.102
						Per	FarmCPU	2.48E-06	30.77	1.734
						Per	BLINK	6.77E-15	12.9	2.287
						SurfA	BLINK	0.000813	38.48	1.008
						TrLngth	BLINK	3.74E-06	17.06	1.151
S09_57233180	C/A	Sb09	57233180	0.108247	RD	ConA	BLINK	1.63E-07	1.27	0.592
S09_55159986	A/G	Sb09	55159986	0.03866	RD	NetA	BLINK	1.82E-10	31.91	− 1.721
						NetA	FarmCPU	0.000542	6.97	− 0.795
						ConA	BLINK	6.84E-24	22.9	2.417
						noRtips	BLINK	7.1E-25	30.28	− 9.428
						Per	BLINK	1.32E-21	19.82	− 2.734
						TrLngth	BLINK	4.88E-10	36.16	− 1.648
S09_57072489	G/A	Sb09	57072489	0.041237	RD	NetA	BLINK	4.12E-05	17.7	− 1.068
						noRtips	BLINK	1.3E-06	12.22	− 3.689
						Per	BLINK	8.28E-09	6.63	− 1.441
						TrLngth	BLINK	0.002743	18.71	− 0.835
S09_1263108	A/G	Sb09	1263108	0.087629	RD	NetA	BLINK	0.000106	3.09	− 0.618
						Per	FarmCPU	0.005951	9.26	− 0.717
						Per	BLINK	8.81E-05	1.23	− 0.724
						SurfA	BLINK	0.003984	18.36	− 0.629
						TrLngth	FarmCPU	0.000233	8.83	− 0.574
*Significant QTL associated with relative differences between the conditions based on the optimal P at 42 DAG*										
S01_1865808	A/T	Sb01	1865808	0.069588	RD	RtW	FarmCPU	0.031027	48.91	0.529
						RtW	BLINK	0.031027	48.92	0.529
S02_60332045	G/A	Sb02	60332045	0.115979	RD	NetA	BLINK	0.003632	0.64	0.488
						SurfA	BLINK	0.001644	2.42	0.525
S02_7792893	G/A	Sb02	7792893	0.048969	RD	NetA	BLINK	1.05E-05	6.73	1.337
S03_72463705	G/T	Sb03	72463705	0.139175	RD	NetA	BLINK	0.000554	1.16	− 0.645
S03_73478713	G/C	Sb03	73478713	0.095361	RD	ConA	FarmCPU	0.00015	9.72	− 0.541
						ConA	BLINK	0.000459	7.29	− 0.481
						noRtips	FarmCPU	4.02E-06	12.11	1.275
						noRtips	BLINK	8.11E-05	1.9	0.918
S04_56655132	A/G	Sb04	56655132	0.39433	RD	TrLngth	BLINK	0.003028	1.54	0.303
S04_6401545	G/A	Sb04	6401545	0.03866	RD	ConA	BLINK	0.000459	8.12	− 0.613
S06_48113932	G/T	Sb06	48113932	0.095361	RD	Per	FarmCPU	0.002405	13.48	− 0.734
S06_51132145	G/T	Sb06	51132145	0.118557	RD	NetA	FarmCPU	5.18E-07	11.61	− 1.242
S06_54299781	G/C	Sb06	54299781	0.134021	RD	NetA	FarmCPU	0.00241	5.27	− 0.873
S07_57211659	C/T	Sb07	57211659	0.036082	RD	Per	FarmCPU	0.000109	6.48	1.438
S09_55159986	A/G	Sb09	55159986	0.03866	RD	ConA	BLINK	6.75E-06	17.03	0.991
						NetA	BLINK	1.71E-13	22.15	− 2.908
						noRtips	BLINK	8.31E-14	39.7	− 3.307
						Per	BLINK	2.05E-10	17.82	− 2.249
						SurfA	BLINK	4E-06	32.35	− 1.601
						TrLngth	BLINK	8.24E-05	15.29	− 0.837
S09_57072489	G/A	Sb09	57072489	0.041237	RD	noRtips	BLINK	4.04E-08	16.93	− 1.803
						SurfA	BLINK	0.000163	15.77	− 1.188
S09_58006574	G/A	Sb09	58006574	0.028351	RD	ConA	FarmCPU	0.00015	38.56	− 0.877
						ConA	BLINK	8.75E-11	13.38	− 1.277
						NetA	BLINK	1.47E-15	18.27	2.574
						noRtips	FarmCPU	0.004405	25.89	0.929
						noRtips	BLINK	8.31E-14	17.93	2.483
						Per	BLINK	1.49E-14	15.42	2.209
						SurfA	BLINK	2.09E-07	14.35	1.682
						TrLngth	BLINK	0.000289	15.78	0.897

Phosphorus deficiency(PD), relative difference (RD),number of root tips (noRtips), total root length (mm) (TrLngth), maximum width (mm) (MaxWdth), network area (mm^2^) (NetA), convex area (mm^2^) (ConA), perimeter (Per), surface area (mm^2^) (SurfA), average root orientation (degrees) (AvOrnt), log10(dry root weight/total plant weight) (RtW:Tw), Fixed and random model Circulating Probability Unification (FarmCPU), Bayesian-information and Linkage-disequilibrium Iteratively Nested Keyway (BLINK), Single Nucleotide Polymorphism (SNP), chromosome number (Chr), SNP position (Pos), minimum allele frequency (MAF), Hartigan and Bartlett Probability Value (H&B.P.Value), Phenotype variance explained (PVE)

### Functional annotation identifies SNPs associated with P starvation

Functional annotation of SNPs within LD blocks revealed their association with numerous genes related to QTL, encompassing a wide range of functions such as those involved in general root growth and development, transferases, hydrolases, transcription factors, DNA repair, and phosphate starvation pathways. Some of these genes displayed either root-specific expression or coexpression with genes in the root coexpression network (Supp. Table 2 and 3) according to the Joint Genome Institute (JGI) Plant Gene Atlas. Examples of such genes associated with QTL for average diameter at 21 DAG included those encoding the protein of unknown function (DUF1138) and the RNA-binding protein SEB4. These genes displayed root-specific expression or coexpression. Meanwhile, genes encoding the V-type H + -transporting ATPase subunit a (ATPeV0A, ATP6N) and Interactor of constitutive active ROPs 3 were associated with perimeter and total root length traits under P deficiency at 42 DAG. Moreover, a SNP S06_48113932, spanning an LD block of 7.1 kb, was associated with genes encoding proteins like the MFS transporter, SP family, solute carrier family 2 (facilitated glucose transporter), member 8 (SLC2A8, GLUT8). This SNP was linked to over six P-related traits in terms of relative differences at both time points. This functional annotation highlights the diverse genetic mechanisms and pathways involved in root growth and development and their relevance to responses under varying nutrient conditions.

Furthermore, we identified 10 QTL distributed across chromosomes Sb02, Sb04, Sb06, and Sb09 associated with proteins that were directly or indirectly involved in phosphate starvation pathway (Table 3). All of which were associated with the relative difference traits, except S02_63147078 and S03_781067, which were identified in response to P deficiency at 42 DAG. Some of the proteins encoded by genes linked to the P starvation response included a Sec14p-like phosphatidylinositol transfer family protein, LORELEI-LIKE-GPI-ANCHORED PROTEIN 1, citrate synthase, plant PDR ABC transporter-associated proteins, glycosyl transferases, among others (Table 3).

**Table 3:** Phosphorus-starvation related QTL under P-deficient conditions and relative differences between optimal P and P deficiency at 21 and 42 days after germination (DAG)

SNP ID	Chr	Pos	Cond	MAF	LD (kb)	DAG	Traits	Gene ID	Description	Protein class/domain
S02_63147078	Sb02	63147078	PD	0.11	7.4	42	AvROrnt, ConA,MaxWdth,NetA,noRtips,Per and TrLngth	Sobic.002G241700	similar to Putative phosphatidylinositol transfer-like protein II	Sec14p-like phosphatidylinositol transfer family protein
								Sobic.002G241900	similar to Putative uncharacterized protein	LORELEI-LIKE-GPI-ANCHORED PROTEIN 1
S02_4706323	Sb02	4706323	RD	0.05	42.2	21	Per	Sobic.002G050000	Predicted protein	DUF1618 domain-containing protein
								Sobic.002G050100	bifunctional inhibitor/seed storage 2 s albumin-like protein-related	Plant lipid-transfer and hydrophobic proteins
S03_781067	Sb03	781067	PD	0.1	0.04	42	Rt:Tw	Sobic.003G008500	similar to Target of myb1-like	VHS domain containing protein family
S04_6401545	Sb04	6401545	RD	0.04	44.3	21	noRtips	Sobic.004G078000	Citrate synthase	Citrate oxaloacetate-lyase ((pro-3S)-CH (2) COO- > acetyl-CoA)
						42	ConA			
S06_51947681	Sb06	51947681	RD	0.06	25.3	21	ConA	Sobic.006G161800	similar to Nodulin-21-like	Ccc1 family
S06_39982967	Sb06	39982967	RD	0.03	92.9	21	NetA	Sobic.006G055600	similar to Heat shock 70 kDa protein	Heat shock protein 70kD, C-terminal domain
S06_48113932	Sb06	48113932	RD	0.1	122	21	Per	Sobic.006G114100	abc transporter g family member 31	Plant PDR ABC transporter associated
S09_57233180	Sb09	57233180	RD	0.11	20.6	21	ConA	Sobic.009G232100	similar to Fasciclin-like protein FLA12	plant-type secondary cell wall biogenesis
								Sobic.009G232200	similar to Fasciclin-like protein FLA15	
S09_55159986	Sb09	55159986	RD	0.04	61.6	21	NetA, noRtips,Per and TrLngth	Sobic.009G202600	similar to Acyltransferase family protein	ancient ubiquitous protein
						42	ConA,NetA,noRtips,Per,SurfA and TrLngth	Sobic.009G202700	weakly similar to Chromosome chr6 scaffold_3	protein aberrant pollen development 1-related
								Sobic.009G203200	similar to Putative pectin methylesterase	pectinesterase
S09_57072489	Sb09	57072489	RD	0.04	24.4	21	NetA,noRtips,Per and TrLngth	Sobic.009G230200	similar to Putative glyoxal oxidase	Galactose oxidase / Beta-galactose oxidase
						42	noRtips and SurfA	Sobic.009G230400	similar to UDP-glucosyltransferase BX8	glucosyl/glucuronosyl transferases

Phosphorus deficiency(PD), relative difference (RD),number of root tips (noRtips), total root length (mm) (TrLngth), maximum width (mm) (MaxWdth), network area (mm^2^) (NetA), convex area (mm^2^) (ConA), perimeter (Per), surface area (mm^2^) (SurfA), and average root orientation (degrees) (AvOrnt), log_10_(dry root weight/total plant weight) (Rt:Tw), chromosome number (Chr), SNP position (Pos), phosphorus treatment condition (Cond), minimum allele frequency (MAF), linkage disequilibrium (LD)

### Haplotype analysis reveals haplotypes with significant effects under P starvation

**Fig. 5:**
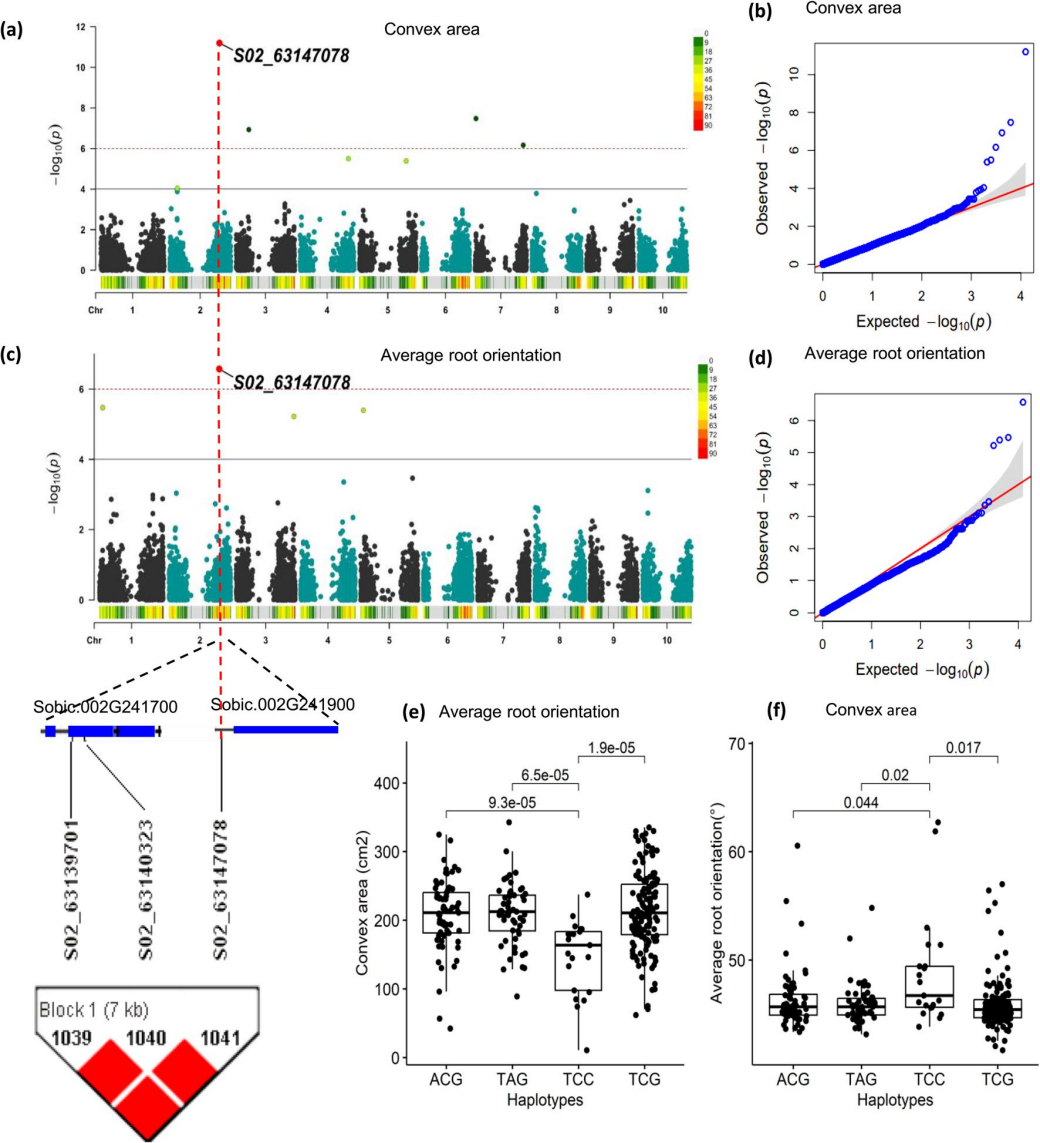
Genome wide association studies and haplotype analysis of a significant QTL at chromosome Sb02 (S02_63147078) associated with Pi starvation at 42 DAG under P-deficient nutrient condition **a** and **b** Manhattan and QQ plot of the SNP marker position and significance associated with convex area trait **c** and **d** Manhattan and QQ plot of the SNP marker position and significance associated with average root orientation trait. **e** A comparison of the three haplotypes to the reference haplotype containing the SNP of interest TCC linked to average root orientation trait **f** A comparison of the three haplotypes to the reference haplotype containing the SNP of interest TCC linked to convex area trait

**Fig. 6:**
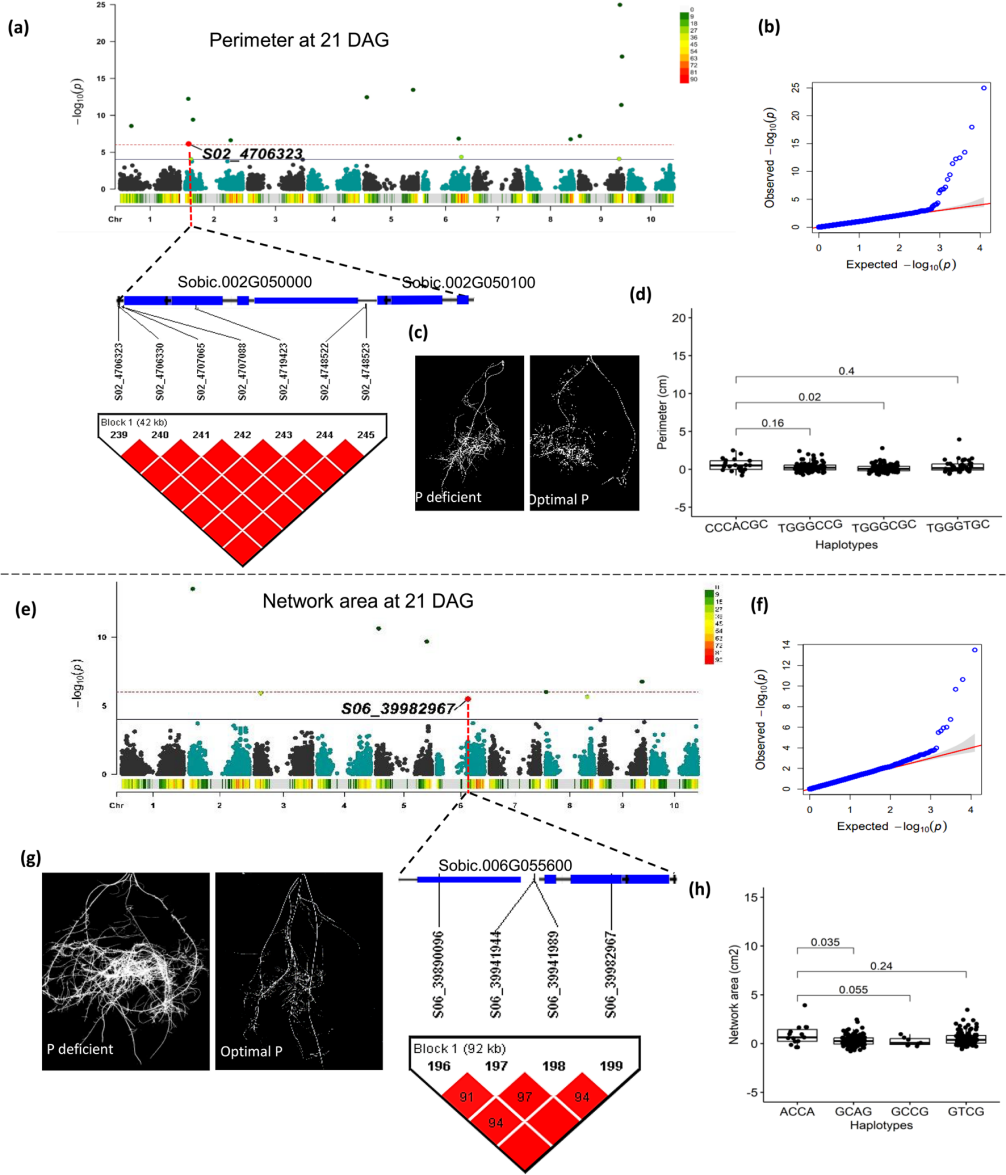
Genome wide association studies and haplotype analysis of two QTL at 21 DAG** a** and **b** A Manhattan plot, QQ plot, and Haploview plot showing significant SNP associated with root perimeter trait at chromosome Sb02 (S02_4706323) and LD block spanning P starvation related gene at 21 DAG **c)** An image showing differences in thickness in sorghum RSA **d** Haplotype effect to the associated trait as shown by a comparison between the reference haplotype to the adjacent haplotypes within the LD block **e** and **f** A Manhattan plot, QQ plot, and Haploview plot showing significant SNP at chromosome Sb06 associated with root network area trait (S06_39982967) and LD block spanning P starvation related genes at 21 DAG **g** An image showing the spread and distribution root exemplifying root network area trait in sorghum **h** Haplotype effect to the associated trait as shown by a comparison between the reference haplotype to the adjacent haplotypes within the LD block

The QTL associated with the P starvation pathway were analyzed further to identify haplotypes within the LD blocks and assess their impact on the traits of interest. Haplotypes spanning three of the ten identified QTL associated with the phosphate starvation pathway (Table 3), displayed significant trait differences (*α* < 0.05) compared to alternative haplotypes within their respective LD blocks. One such haplotype was TCC, which was linked to the alternative allele C of marker S02_63147078 at 42 DAG. This haplotype showed significant differences in means as compared to the three alternative haplotypes ACG, TAG, and TCG for the associated traits (Supp. Table 4 and Supp. Figure 10). Traits such as NetA, ConA and TrLngth exhibited negative significant differences for this haplotype, while AvROrnt displayed a positive significant difference as compared to alternative haplotypes within the block (Fig. 5 and Supp. Figure 10). Additionally, two QTL (S02_4706323 and S06_39982967) encompassed by four haplotypes each within an LD block showed significant positive differences in means (*α* < 0.05) within the block for their corresponding traits at 21 DAG (Fig. 6a and e). For instance, haplotype CCCACGC spanning a QTL on chromosome Sb02, showed a positive significant difference as compared to alternative haplotypes for the root perimeter trait (Fig. 6d). Likewise, haplotype, GCCG, which encompassed a QTL on chromosome Sb06 showed a positive significant difference as compared to alternative haplotypes for the root network area trait (Fig. 6h and Supp. Table 4).

## Discussion

### Response of *sorghum* RSA to P starvation

Physiological adaptations of RSA during early growth stages play a crucial role in determining the later successful establishment of crops, especially under stressful conditions. Soil P deficiency problem has prompted breeders to prioritize the development of P-efficient crops for both current and future agro-ecological systems (Han et al. 2022). In this study, we investigated the genetic response and adaptation of sorghum RSA to P starvation conditions in a hydroponic system at two different time points. Hydroponic paper systems and semi-hydroponic experiments have been used previously to discover molecular markers associated with P starvation in maize (Liang et al. 2023), wheat (Dharmateja et al. 2021), and sorghum (Tishchenko et al. 2020). As a result, many RSA traits can now serve as valuable resources for early P deficiency detection in plants.

Plants typically survive P starvation conditions by developing a combination of two or more RSA traits (Lynch 2011). RSA traits that signify root density and distribution, such as total root length, surface area, volume, and root tips, have been linked to adaptation to low P conditions in bread wheat and sorghum (Dharmateja et al. 2021; Gladman et al. 2022; Liang et al. 2023). In our study, similar traits exhibited significant differences under P-deficient conditions, as indicated by high CV and significant differences in means (Table 1). According to Gladman et al. (2022), the overall increase in volume and surface area of sorghum roots directly corresponded to a significant increase in lateral root growth. The significant interaction between genotypes and nutrient supply for these traits, combined with high heritability, clearly demonstrated the intrinsic impact of P treatment within the examined population, which will enable high selection efficiency at early stages of growth. The broad-sense heritability, ranging from 0.3 to 0.76, was consistent with previous reports in sorghum (Parra-Londono et al. 2018; Bernardino et al. 2019).

Positive correlations observed between P-related traits confirmed the collective influence of RSA on root growth and development, particularly under P starvation. The strong positive correlations between dry shoot weight (*r* > 0.7) and traits such as SurfA and ConA, contrasting with the negative correlation observed with AvDim, which had a minimal impact on P starvation conditions, highlighted the subtle responses of plants to varying P levels. This was further confirmed by a strong positive correlation (*r* > 0.6) between root surface area and total root length, alongside a weaker correlation (*r* > 0.45) with root diameter. Thus, under P starvation sorghum RSA development shifts toward longer and thinner roots at the expense of shorter and thicker roots. Both thick-short and thin-long RSA increases the surface for efficient uptake of the deficient nutrient. However, the preference toward thinner (as relating to root diameter) and longer roots observed emphasizes the explorative nature of sorghum which is a typical topsoil foraging response by plants under P deficiency.

Besides the absolute means of RSA traits, the relative differences between low and high P conditions, which reflects the response of sorghum to P starvation, have also provided valuable insights for molecular resources in RSA breeding for arable crops (Lopez et al. 2023). In this study, significant magnitudes of change (> 0) were observed in all P-related RSA traits, except for AvROrnt, suggesting the adaptability of plants to P scarcity. Our results align with previous research by Lopez et al. (2023), demonstrating higher magnitudes of change in RSA traits such as root length, specific root length, and root-to-shoot ratio in monocots under low P conditions. Collectively, our results underline the evidence that RSA is adaptable to changes in P levels and a focus on particular traits will enable crop improvement and resilience under P conditions. The identified P-related traits aligning with previous research provide a basis for further exploration of RSA dynamics that can be integrated in sorghum breeding programs to enhance crop adaptation to fluctuating P levels and ensure sustainable productivity.

### Classification of RSA types across time points and conditions

The root system architecture diversity in contrasting P conditions, provides clarity on the impact of P starvation within the population. Composite classifications of RSA types based on PCA, biplot analysis, and cluster analysis have been achieved in many crops (Morais De Sousa et al. 2012; Reddy et al. 2020; Duresso et al. 2023). This approach serves as a starting point for selecting genotypes with better adaptation to low P conditions (Reddy et al. 2020). In our study, we identified four distinct clusters within the diversity panel at both time points and P conditions (Fig. 4). The primary contributing factors to these clusters were the genetic diversity within the population and P treatment. Many of the aforementioned P-related RSA traits, such as Per, TrLngth, NetA, SurfA, and the noRtips, were major contributors to PC1 at all conditions and time points. Similar traits have been identified as major contributors to root diversity under low P conditions in mung bean (*V.radiata*) (Reddy et al. 2020), maize (Morais De Sousa et al. 2012), and sorghum (Parra-Londono et al. 2018). However, the small differences observed between the two time points can be attributed to the pronounced adaptations to P deficiency at 42 DAG and the corresponding tradeoffs among the traits. Alternatively, while RSA traits like AvDim, Soldty, and AvOrnt tended to positively contribute to PC2 at both time points and conditions, Soldty and AvDim exhibited higher correlations at 42 DAG than at 21 DAG (Fig. 4). Altogether, this was an indication of diverse adaptability progress within the population characterized by an earlier stress and later response to P starvation.

The adaptive response to P treatment was confirmed by our classification that showed a significant change at 42 DAG as compared to 21 DAG. This was evident by the nearly identical clustering observed between P-deficient and optimal P conditions at 21 DAG (Supp. Figure 8). In addition to the distinct root types identified as highly branched, restricted branched, coarse, and restricted at 42 DAG (Degens 1997), the significant increase in root density and distribution can be attributed not only to time but also to the P treatment (Fig. 4e). The extreme clusters 3 and 4 under P deficiency, at 21 and 42 DAG, respectively, suggest their adaptability to low P conditions when compared to most individuals in clusters 1 and 2. This type of classification has also been adopted in other crops (Morais De Sousa et al. 2012; Reddy et al. 2020).

### Significance of QTL associated with P starvation pathways

During P starvation, plants exhibit adaptive responses, similar to other stressful conditions. These adaptations can manifest diversely. In typical P-deficient conditions, plants adapt by altering their root systems, reconfiguring nutrient translocation among organs, or modifying the cellular environment. Many of these mechanisms involve transporters, transcription factors, and hydrolytic enzymes, not only functioning at the cellular level but also having effects spanning tissues and organs. In this study, we identified several QTL associated with genes encoding proteins linked to root-specific expression, as detailed in the JGI Plant Gene Atlas (McCormick et al. 2018). Some of these proteins include DUF1138a, protein of unknown function, the RNA-binding protein SEB4, the V-type H + -transporting ATPase subunit a (ATPeV0A, ATP6N), and an interactor of constitutive active ROPs, among others. These QTL-linked markers represent new information which warrants further analysis to determine the potential involvement of nearby genes in phosphate-related pathways.

More specifically, some of the QTL associated with genes in our study encoded proteins involved in previously identified phosphate-starvation pathways (Table 3). For instance, a transcriptional study found that a gene encoding a nodulin 21-like protein was downregulated under iron deficiency but upregulated under P deficiency in rice (*O. sativa*) (Takehisa et al. 2013) and apple trees (*Malus domestica*) (Valentinuzzi et al. 2019). Similar studies also confirmed the upregulation of the ABC transporters g family in Stylo (*S.guianensis*) roots and an increase in citrate synthase expression in Chinese fir (*C. lanceolata*) roots under P-deficient conditions (Chen et al. 2021; Wu et al. 2023). Another phosphate-starvation pathway-related protein identified in our study was a pectin methylesterase. Its expression was linked to increased cellular P reuse in rice and *Arabidopsis* (Uno et al. 2017; Huang et al. 2022; Qi et al. 2022). This enzyme was also observed to enhance the activities of *PHOSPHORUS-TRANSPORTER-2* under low nitrate conditions and promote the growth of root hairs and surface area in rice (Zhu et al. 2018; Huang et al. 2022). At chromosome Sb03, we identified a QTL associated with the root-to-total plant weight trait linked with the Sb03g000910 gene, responsible for encoding the Target of *MYB1*-like (*TOM1*) protein with a potential post-translation clathrin binding function during P deficiency. Previously, the upregulation of *MYB1* had been correlated with P deficiency (Gu et al. 2017). However, according to this study, *MYB1* expression remained constant irrespective of the P levels, yet its post-translational regulation varied, suggesting an unknown complex mechanism by which it regulates plant’s response to P deficiency. The *TOM1* gene, identified in our study, is situated 4317 base pairs away from a *MYB1* gene and 249 base pairs adjacent to the translation initiation factor 3 subunit L (*EIF3L*) gene (McCormick et al. 2018). This proximity could suggest a probable post-translational regulation on *MYB1* expression under conditions of P starvation.

Additionally, we identified QTL associated with genes encoding an Acyltransferase family protein (glycerol-3-phosphate acyltransferase 1, *GPAT1*), a member of the lipid transmembrane remodeling protein family that initiates the synthesis and expression of LYSOPHOSPHATIDIC ACID ACYLTRANSFERASE 2 (*LPAT2*), hence stimulating enhanced root growth under P deficiency (Angkawijaya et al. 2017). This protein family may also participate in recycling inorganic Pi under P deficiency in rice (Du et al. 2021). We also identified an aberrant pollen development 1-related protein (Zinc/RING finger domain; C3HC4-type RING finger), which is known to be expressed under low phosphorus conditions in maize (Li et al. 2012). E3 ligases, to which this protein belongs, form an intrinsic and well-coordinated transport system that facilitates Pi movement within a complex, coordinated phosphorylation pathway (Ye et al. 2018; Sun et al. 2022). While these genes and proteins provide preliminary resources for the genetic basis of P-response traits in sorghum, corresponding quantitative experiments are needed to comprehensively understand the molecular mechanisms underlying these responses.

### Effects of haplotypes associated with P starvation

Haplotype analysis revealed closely related haplotypes within the LD block associated with genes encoding proteins relevant to P starvation, and exhibiting significant differences for their respective traits. For instance, a SEC-14-like phosphatidylinositol protein, previously involved in converting phospholipids into non-phosphorus-containing galactolipids essential for root growth in *A. thaliana* under P starvation (Nakamura et al. 2009), was one such protein with four haplotypes showing significant difference for seven traits in this study. Likewise, a rice gene *OsSNDP1* (*Oryza sativa* Sec14-nodulin domain protein), was also implicated in root hair growth, albeit under water-logging conditions (Huang et al. 2013). Additionally, the sorghum gene *Sobic.002G241900* within the same LD block encoding a phosphatidylinositol transfer-like protein II was also identified in this study (Table 3 and Supp. Table 4). This gene encodes a LORELEI-LIKE-GPI-ANCHORED PROTEIN 1, previously correlated with immune responses related to a potential increase in Pi uptake in *A. thaliana* (Shen et al. 2017; Tang et al. 2022). The QTL associated with the genes above exhibited significant differences in means at the haplotype level, with contrasting effects on average root orientation as compared to convex area (Fig. 5e and f) and five other traits (Supp. Figure 10). The significant increase in average orientation suggests greater root hair development, ultimately affecting overall root growth. In contrast, the significant decrease in convex area implies that roots reallocating their resources toward producing more lateral roots and root hairs that may compromise the general development of primary roots.

Furthermore, the other haplotype groups associated with relative differences in perimeter (Fig. 6d) and network area (Fig. 6h) showed significant variations compared to at least one other haplotype within the LD block at 21 DAG. The haplotype linked to the perimeter encoded two proteins: a protein of unknown function (DUF1618) and a bi-functional inhibitor/seed storage 2S albumin-like protein-related encoded by the *DEFECTIVE IN INDUCED RESISTANCE 1* (*DIR1*) gene. The *DIR1* protein is one of the lipid-binding transmembrane proteins implicated in the translocation of long-distance tissue signals during system acquired resistance (Champigny et al. 2011). The haplotype associated genes and encoded proteins identified here provides novel genetic resources for early detection of P-response in sorghum. However, validation will be essential for further confirmation of the associated genes.

## Conclusion

In conclusion, our findings suggest that P starvation has a more pronounced impact on the sorghum root system during earlier growth stages (up to 3 weeks after sowing) compared to later stages (3 to 6 weeks). The adaptive responses exhibited by sorghum roots involve an increase in root size and distribution, rather than alterations in root orientation and thickness. These genetic adaptations are noteworthy at the population level, leading to the distinct classification of different root system ideotypes, especially at 42 DAG. Moreover, exposure to P starvation revealed associations with both new and previously described genes that are directly or indirectly associated with the phosphate starvation pathway in sorghum. Distinct traits revealed a high plasticity of sorghum roots under contrasting P availability, while GWAS identified P-responsive QTL with general effects on the genetic architecture of the root system. The genetic and physiological adaptations unveiled in this study will improve our understanding of genetic mechanisms of root system plasticity under P deficiency and expand available resources for sorghum breeding.

## Supplementary Information

Below is the link to the electronic supplementary material.

Supplementary file1 (PDF 1850 KB)

Supplementary file2 (XLSX 1211 KB)

## Data Availability

Data and plant materials used in this study are available from the authors on request.
