# Supplementary material for: Early exposure to phosphorus starvation induces genetically determined responses in Sorghum bicolor roots
Source: Theor Appl Genet. 2024 Sep 11;137(10):220. doi: 10.1007/s00122-024-04728-4 (PMC11390786; doi:10.1007/s00122-024-04728-4)
Supplement: Supplementary file 1 — Supplementary file1 (PDF 1850 KB) [file 122_2024_4728_MOESM1_ESM.pdf]

Early phosphorus starvation induces genetically determined responses in *Sorghum bicolor* roots

Theoretical and applied Genetics, Supplementary figures

Mikwa O. Erick[1*], Benjamin Wittkop[1], Steffen M. Windpassinger[1], Sven E. Weber[1], Dorit Ehrhardt[1] and Rod J. Snowdon[1]
[1]Department of Plant Breeding, Justus Liebig University Giessen, Germany

Correspondence: Erick Owuor Mikwa, erick.owuor.mikwa@ag.uni-giessen.de

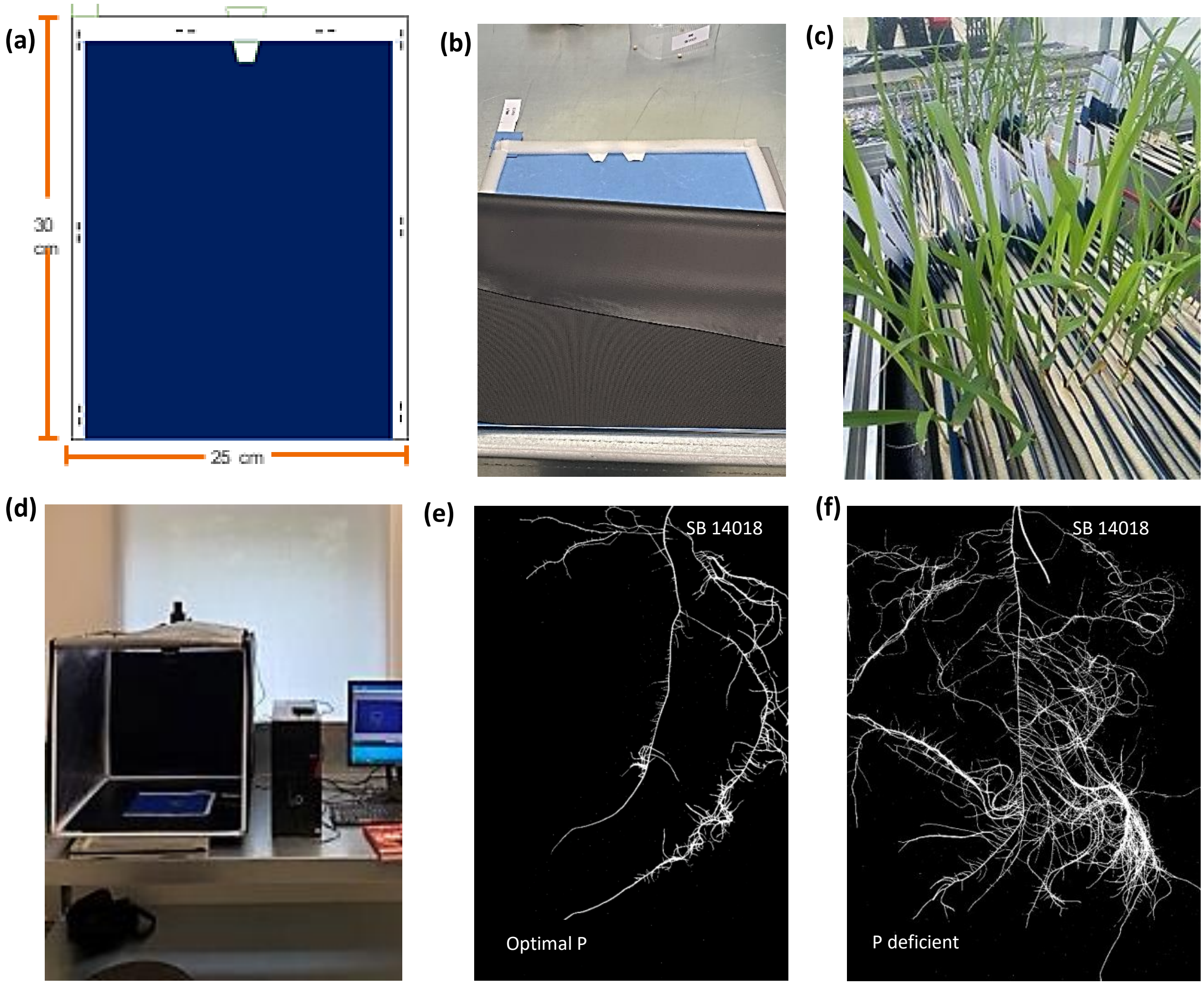

Fig. 1 a) Visual dimensions and details of growth paper pouch **b)** A complete assembly of the paper pouch including black polythene cover ready for sowing. **c)** Paper pouches with sorghum plants at 21 days after sowing **d)** Image capturing set up showing light box, with a camera on a stand and a computer installed with the camera software **e)** and **f)** Genotype SB 14018 showing visual increase in root density and distribution in between optimal P and P deficient nutrient conditions, respectively

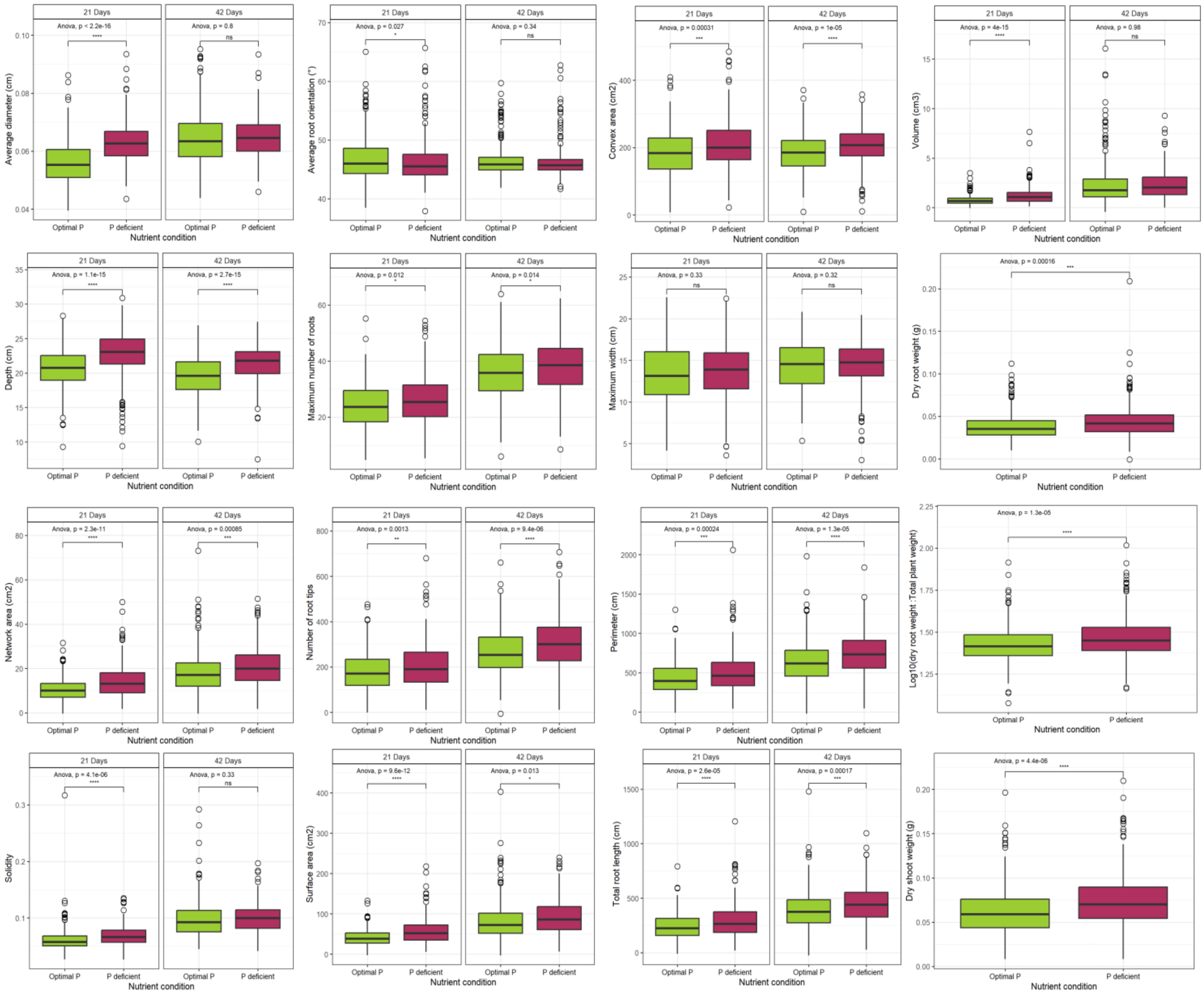

Fig. 2 Boxplots showing comparisons of means between optimal P and P deficient conditions at both 21 and 42 DAG. The overall means were calculated based on the adjusted means as estimated using equation [3]

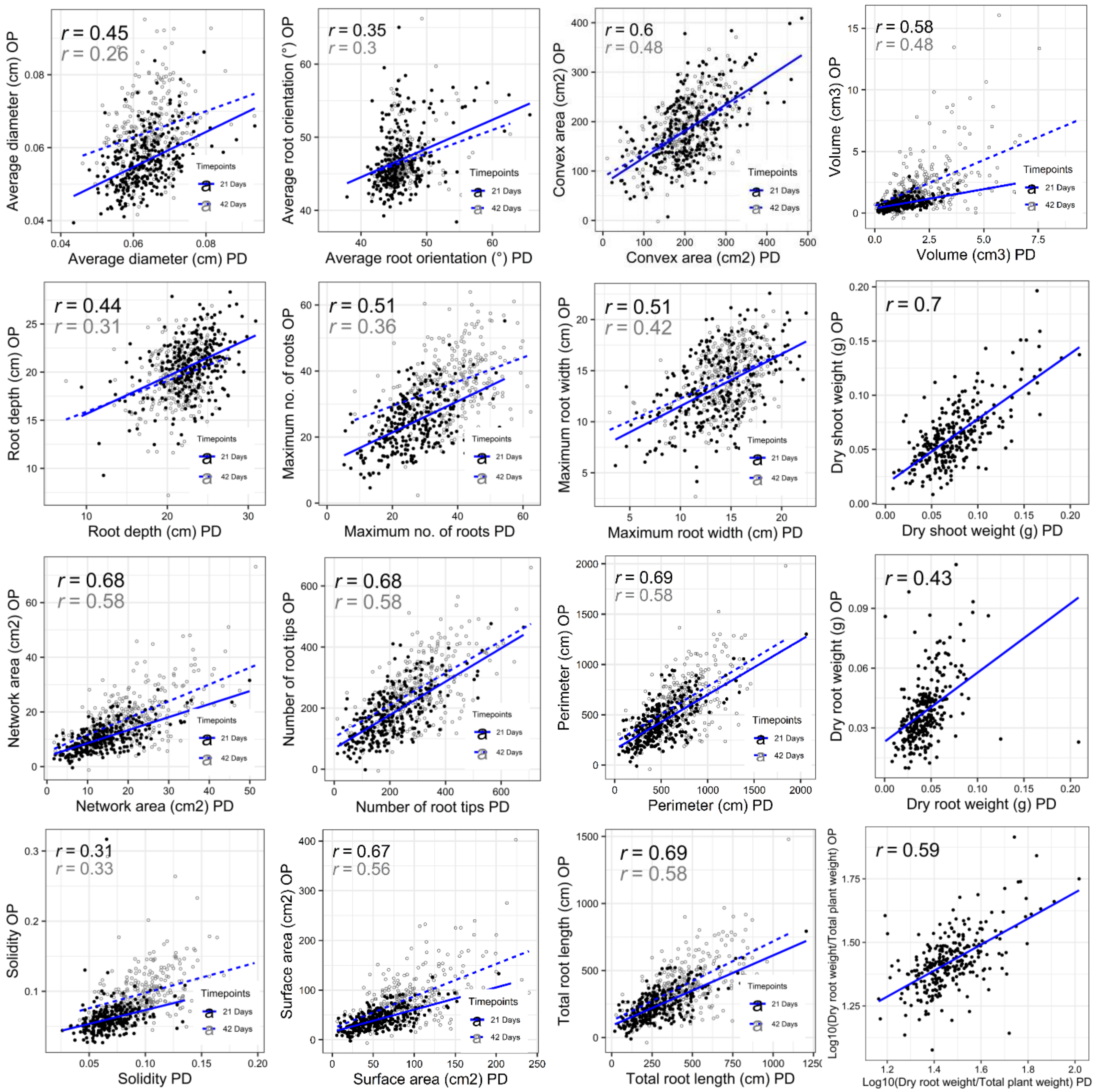

Fig. 3 Pearson's correlation per trait across time points and conditions. Each figure represents a single trait and correlations were obtained from adjusted means as estimated using equation [3]

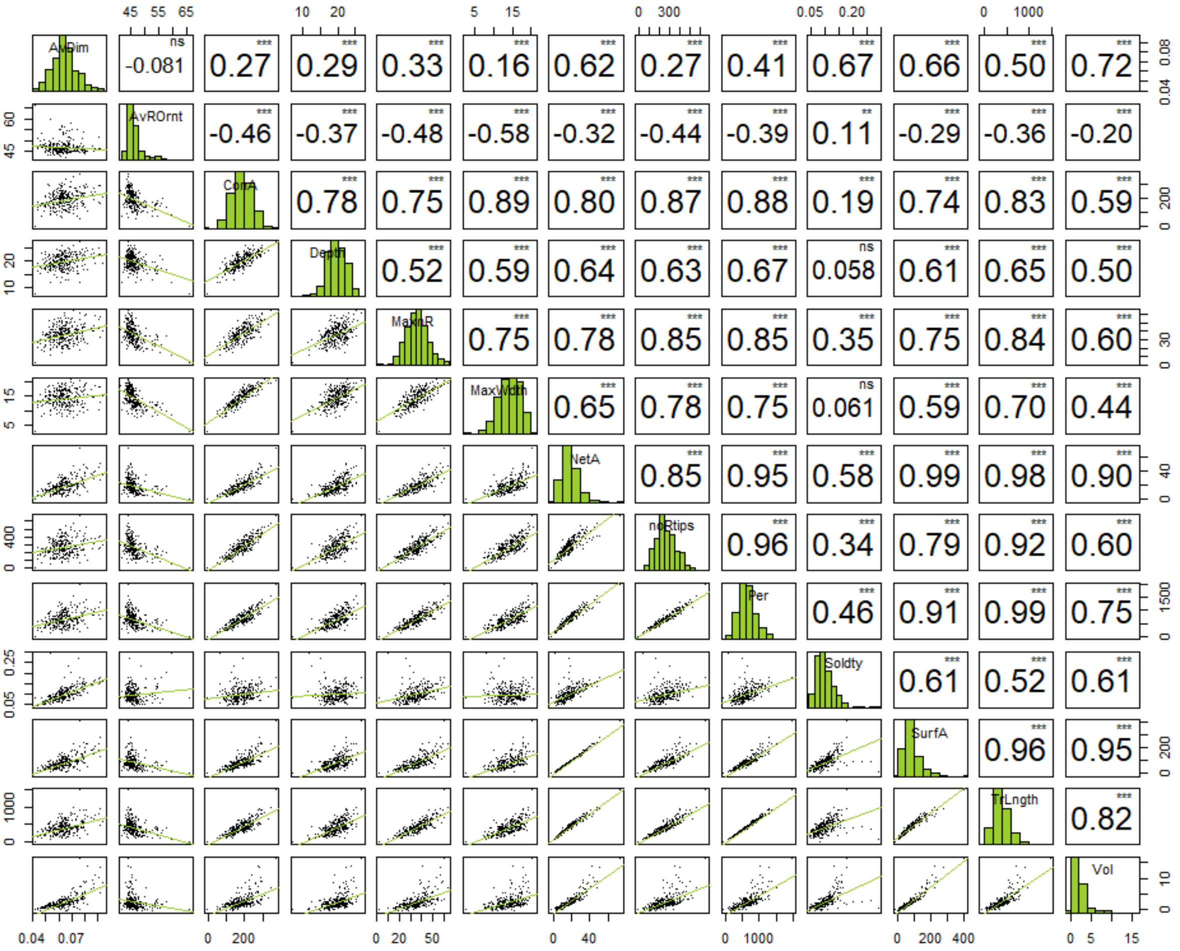

Fig. 4 Pearson's correlation matrix showing correlation of RSA traits in sorghum under optimal nutrient conditions 21 days after germination in a control greenhouse hydroponic system. Significance Codes: less than 0.01 '***'; greater than 0.01 '**'; greater than 0.1 'ns'. Trait codes: average diameter (AvDim); average root orientation (AvOrnt); convex area (ConA); maximum number of roots (MaxnR); maximum width (MaxWdth); network area (NetA); number of root tips (noRtips); perimeter (Per); solidity (Soldty); surface area (SurfA); total root length (TrLngth); volume (Vol)

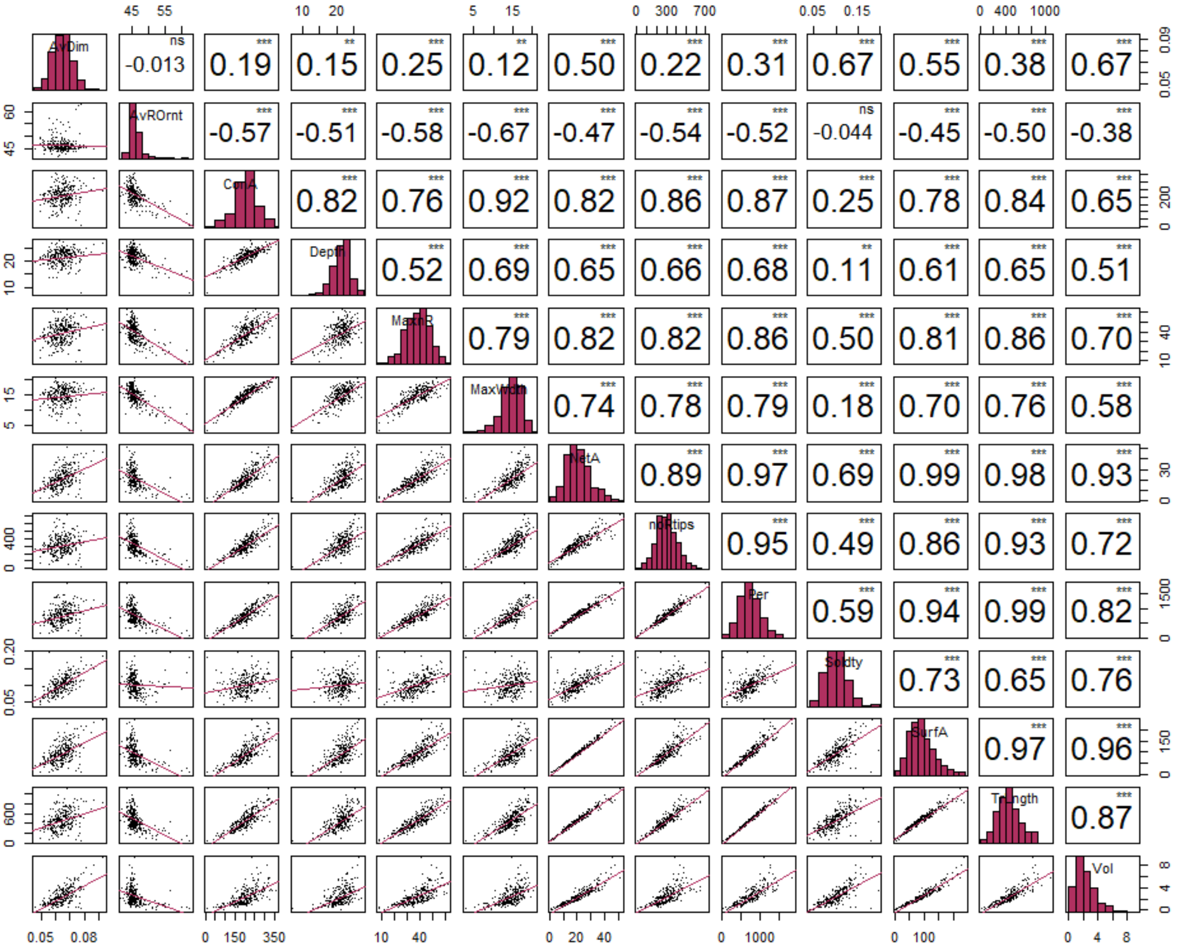

Fig. 5 Pearson's correlation matrix showing correlation of sorghum RSA traits under phosphorus deficient nutrient condition 21 days after germination in a control greenhouse hydroponic system. Significance Codes: less than 0.01 '***'; greater than 0.01 '**'; greater than 0.1 'ns'. Trait codes: average diameter (AvDim); average root orientation (AvOrnt); convex area (ConA); maximum number of roots (MaxnR); maximum width (MaxWdth); network area (NetA); number of root tips (noRtips); perimeter (Per); solidity (Soldty); surface area (SurfA); total root length (TrLngth); volume (Vol)

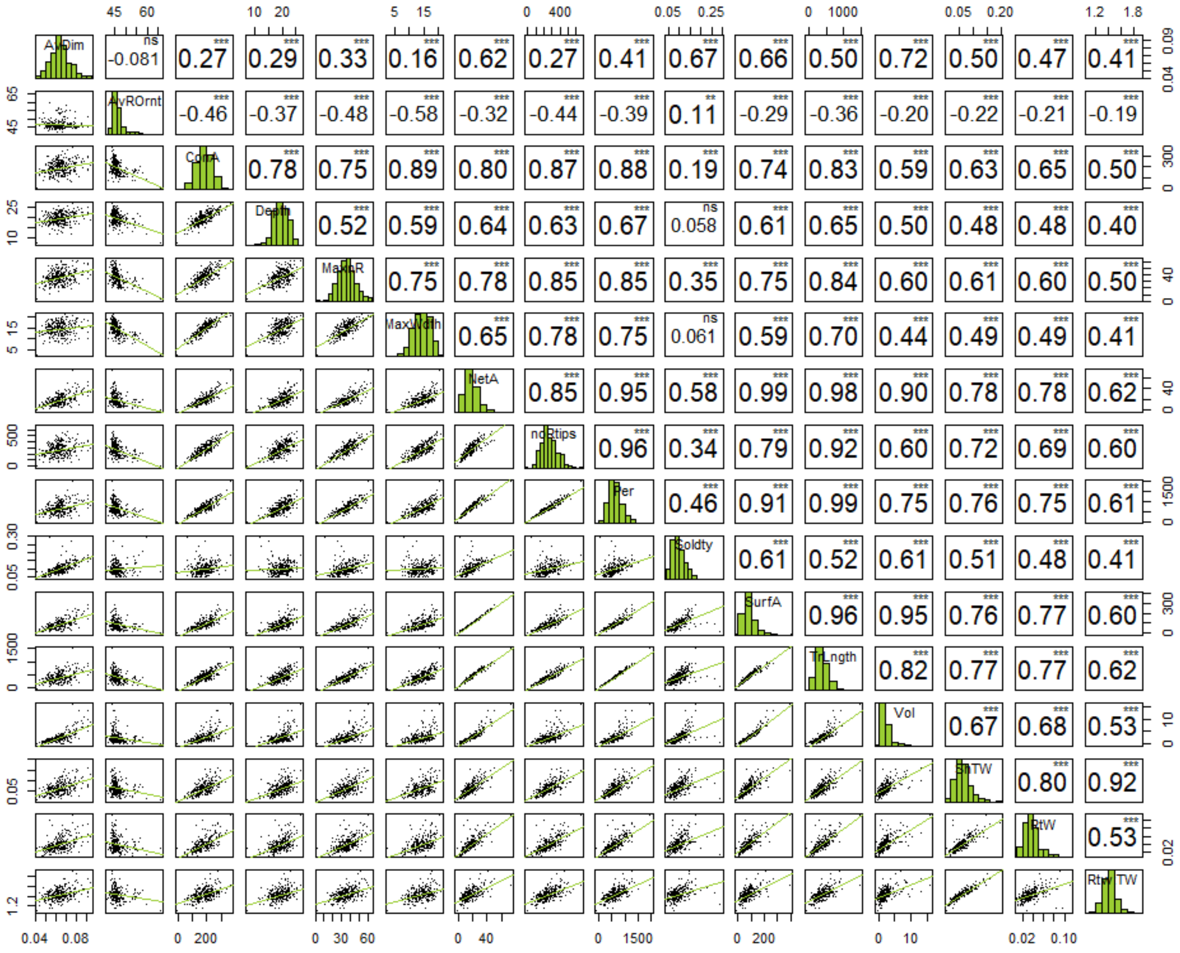

Fig. 6 Pearson's correlation matrix showing correlation of RSA traits in sorghum under optimal nutrient conditions 42 days after germination in a control greenhouse hydroponic system. Significance Codes: less than 0.01 '***'; greater than 0.01 '**'; greater than 0.1 'ns'. Trait codes: average diameter (AvDim); average root orientation (AvOrnt); convex area (ConA); maximum number of roots (MaxnR); maximum width (MaxWdth); network area (NetA); number of root tips (noRtips); perimeter (Per); solidity (Soldty); surface area (SurfA); total root length (TrLngth); volume (Vol); dry shoot weight (ShtW); dry root weight (RtW); Log10(dry root weight/Total plant weight) (Rtw/Tw)

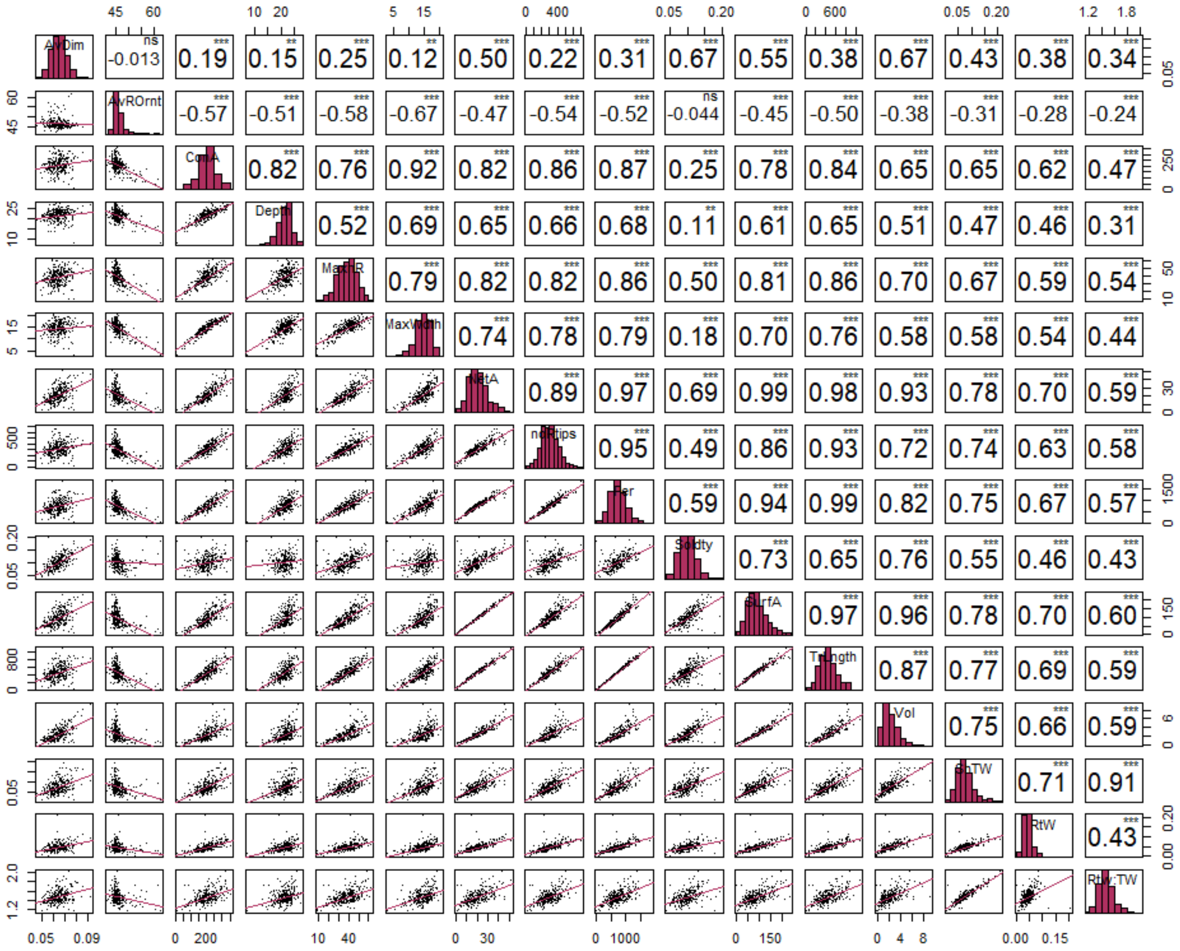

Fig. 7 Pearson's correlation matrix showing correlation of sorghum RSA traits under phosphorus deficient nutrient condition 42 days after germination in a control greenhouse hydroponic system. Significance Codes: less than 0.01 '***'; greater than 0.01 '**'; greater than 0.1 'ns'. Trait codes: average diameter (AvDim); average root orientation (AvOrnt); convex area (ConA); maximum number of roots (MaxnR); maximum width (MaxWdth); network area (NetA); number of root tips (noRtips); perimeter (Per); solidity (Soldty); surface area (SurfA); total root length (TrLngth); volume (Vol); dry shoot weight (ShtW); dry root weight (RtW); Log10(dry root weight/Total plant weight) (RtW/Tw)

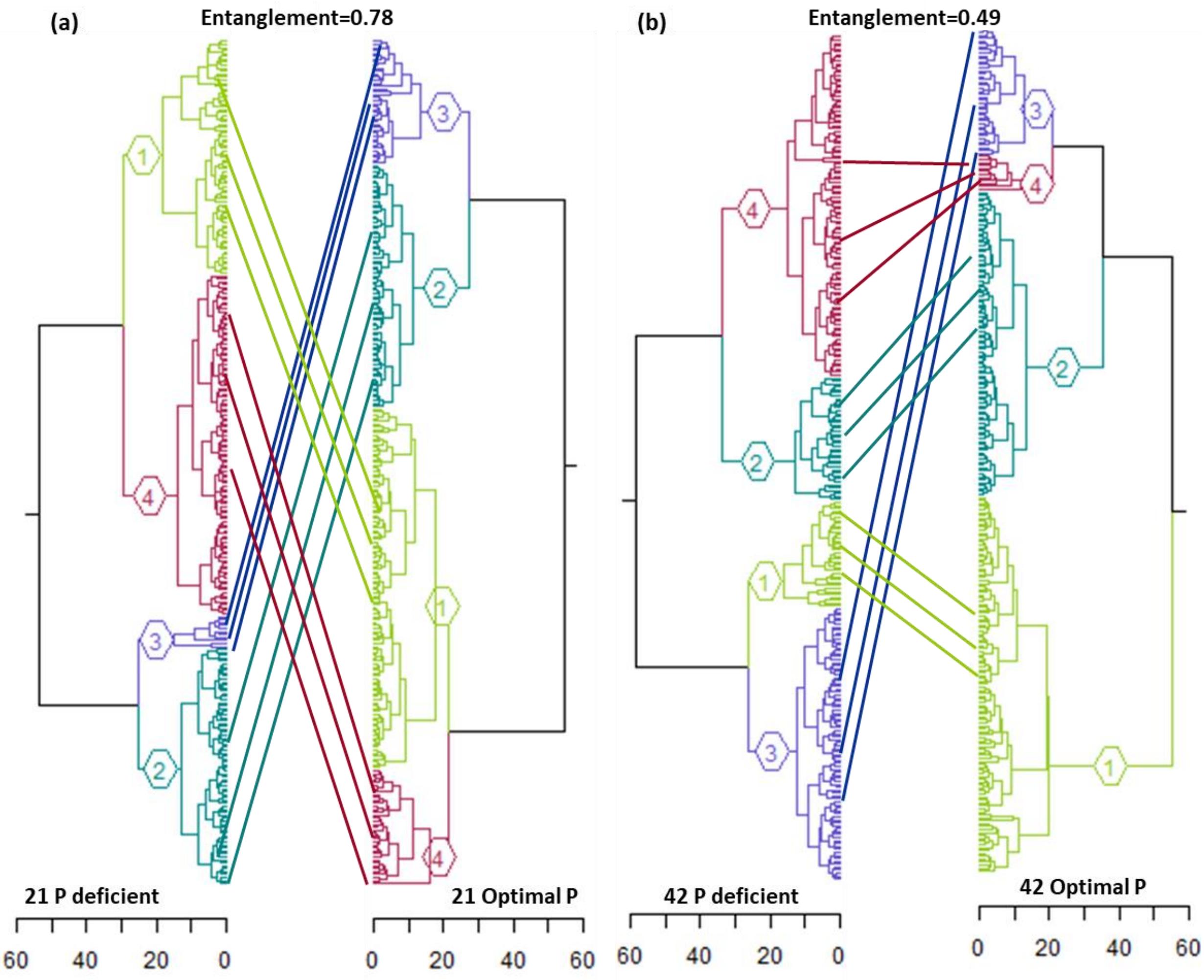

Fig. 8 Tanglegram showing similar and distinct clusters between optimal P and P deficient conditions **a)** at 21 DAG and **b)** 42 DAG

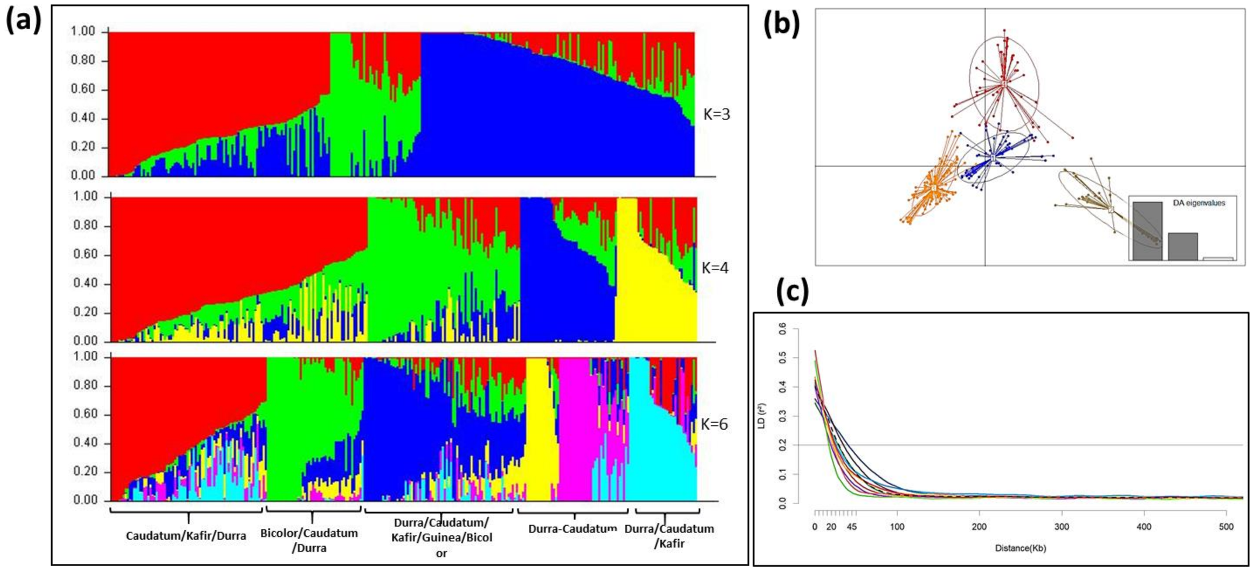

Fig. 9 a) Clusters from STRUCTURE software showing admixture from optimal K=3, K=4 and K=6. The optimal cluster (K) was determined based on the rate of change in the log probability of data (Ln [P (K)]) across the different K values according to the Evanno method (Evanno et al., 2005) **b)** Discriminant Analysis of Principal Components (DAPC) graph showing four genetic subgroups with the population **c)** Linkage Disequilibrium (LD) in all the ten chromosomes and an average decay at about 45 kb for the SNP panel used in the population

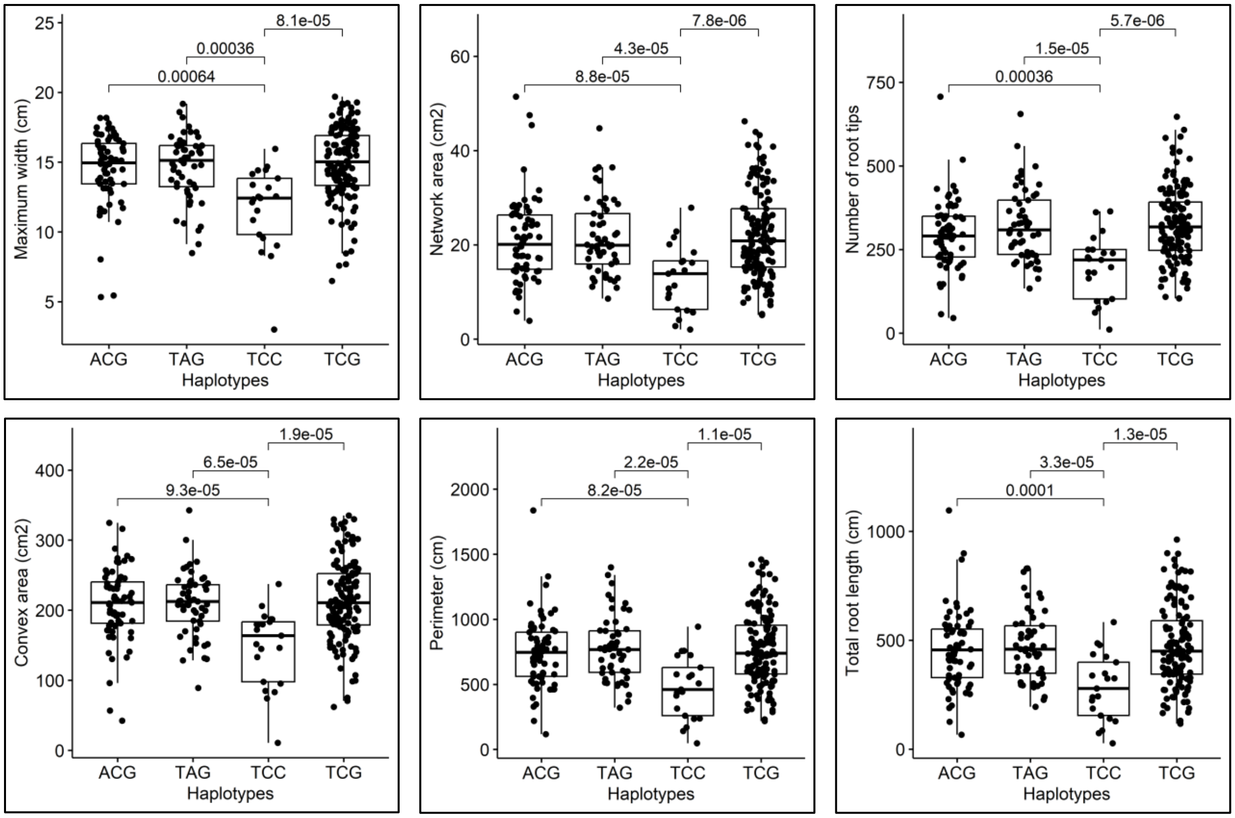

Fig. 10 A comparison of haplotype effect of three haplotypes to the haplotype containing the SNP of interest (TCC) associated with maximum width, network area, number of root tips, convex area, perimeter and total root length traits in sorghum root.
